# Optoception: Perception of Optogenetic Brain Perturbations

**DOI:** 10.1523/ENEURO.0216-22.2022

**Published:** 2022-06-27

**Authors:** Jorge Luis-Islas, Monica Luna, Benjamin Floran, Ranier Gutierrez

**Affiliations:** 1 Laboratory of Neurobiology of Appetite. Department of Pharmacology, Center for Research and Advanced Studies of the National Polytechnic Institute, CINVESTAV, Mexico City, Mexico; 2 Department of Physiology, Biophysics, and Neurosciences, Center for Research and Advanced Studies of the National Polytechnic Institute, CINVESTAV, Mexico City, Mexico, 07360

**Keywords:** brain manipulations, interoception, optogenetics, self-perception

## Abstract

How do animals experience brain manipulations? Optogenetics has allowed us to manipulate selectively and interrogate neural circuits underlying brain function in health and disease. However, little is known about whether mice can detect and learn from arbitrary optogenetic perturbations from a wide range of brain regions to guide behavior. To address this issue, mice were trained to report optogenetic brain perturbations to obtain rewards and avoid punishments. Here, we found that mice can perceive optogenetic manipulations regardless of the perturbed brain area, rewarding effects, or the stimulation of glutamatergic, GABAergic, and dopaminergic cell types. We named this phenomenon optoception, a perceptible signal internally generated from perturbing the brain, as occurs with interoception. Using optoception, mice can learn to execute two different sets of instructions based on the laser frequency. Importantly, optoception can occur either activating or silencing a single cell type. Moreover, stimulation of two brain regions in a single mouse uncovered that the optoception induced by one brain region does not necessarily transfer to a second not previously stimulated area, suggesting a different sensation is experienced from each site. After learning, they can indistinctly use randomly interleaved perturbations from both brain regions to guide behavior. Collectively taken, our findings revealed that mice’s brains could “monitor” perturbations of their self-activity, albeit indirectly, perhaps via interoception or as a discriminative stimulus, opening a new way to introduce information to the brain and control brain-computer interfaces.

## Significance Statement

We propose that most optogenetic brain manipulations may serve as a conditioned cue to guide behavioral decisions and learning, probably using a variety of either interoception, percepts, or other sensory/motor responses evoked by perturbing distinct brain circuits. Further research should uncover whether optoception is a fundamental property everywhere in the brain and unveil its underlying mechanisms.

## Introduction

The brain can sense and integrate signals arising from inside the body, monitoring the status of the internal milieu in a process named interoception (Craig, 2002; Khalsa and Lapidus, 2016). A more inclusive definition; interoception is the perception of the state of the body (Ceunen et al., 2016). This process which is not restricted to visceral stimuli, occurs both consciously and nonconsciously and comprises all body organs, including the brain (Hölzl et al., 1996; Craig, 2002). This opens the possibility that the brain senses its own perturbations. The two main techniques to perturb the brain are either electrical or optogenetic stimulation. It is well established that electrical stimulation of various areas can produce a cornucopia of behavioral changes (Verrier et al., 1975; Di Scala et al., 1987; Romo et al., 1998; Guo et al., 2015; Wu et al., 2016; Mazurek and Schieber, 2017). For example, Doty (1965) found that monkeys can perceive and report the electrical stimulation from most brain areas assayed, covering 38 cortical and 31 subcortical regions (Doty, 1965). Monkeys can also report electrical stimulation from the spinal cord (Yadav et al., 2021). Also, Romo et al. (1998) demonstrated that monkeys perceived the frequency of the electrical stimulation delivered in the somatosensory (S1) cortex as if their fingertip was being touched (Romo et al., 1998). More recently, Mazurek and Schieber “injected instructions” directly into the monkeys’ premotor cortex by varying different parameters of the electrical stimulation to solve an arm reaching task (Mazurek and Schieber, 2017; for another interpretation, see Lebedev and Ossadtchi, 2018). Likewise, rats can detect an infrared light source (Thomson et al., 2013) or solve a maze only using electrical stimulation to guide behavior (Wu et al., 2016).

A variety of behavioral effects are also induced by optogenetics: the pioneering work of Daniel Huber and Karel Svoboda has shown that a brief optogenetic perturbation of cortical neurons in the barrel cortex could drive perceptual decisions and learning. Specifically, they showed that mice could learn to report optogenetic cortical perturbations (Huber et al., 2008). Subsequent studies further elaborated on the nature of sensory percepts evoked by optogenetics, ranging from a whisker-like sensation (Sachidhanandam et al., 2013), an illusory pole touch (O’Connor et al., 2013), to touching a virtual wall (Sofroniew et al., 2015), but only when mice were whisking, suggesting the need for a sensory-motor integration for perception (O’Connor et al., 2013). Intriguingly, the “optogenetic programming of behavior” further improved learning performance (Sachidhanandam et al., 2013), surpassing the natural sensory stimulation of a single whisker (Sofroniew et al., 2015). Thus, optogenetic perturbations in the barrel cortex seem to induce more than a “pure” somatosensory percept but rather an exacerbated, perhaps an artificial sensation that can be readily perceived. Impressively, even a sparse activation of ∼14 randomly selected pyramidal neurons in the barrel cortex was sufficient for mice to induce a “conscious” perception of cortical activity. After training, fewer neurons were required to induce perception, suggesting the induction of synaptic plasticity (Dalgleish et al., 2020). Surprisingly, optogenetic stimulation of motor-related D1 striatal spiny neurons can also readily replace previously trained whisker sensory stimuli, suggesting that mice can report “motor-related signals” as if they were experienced a whisker-like stimulation (Sippy et al., 2015). Similar results have been obtained in other sensory cortices. The holographic optogenetic activation of neuronal ensembles selectively tuned to visual stimuli (e.g., horizontal lines) elicited a perception as if mice saw a horizontal line (Carrillo-Reid et al., 2019; Marshel et al., 2019). Likewise, electrical stimulation can also induce olfactory (Vetere et al., 2019), auditory (Campolattaro et al., 2007; Halverson and Freeman, 2010), or visual sensations (Halverson et al., 2009), depending on the brain region perturbed. However, in all these studies, it is usually assumed that the evoked experiences mimic the physiological function and sensory qualia ascribed to the perturbed neuronal circuit (Guo et al., 2015; Marshel et al., 2019; Gill et al., 2020). All in all, these data suggest that subjects could be aware of brain perturbations in sensory regions.

However, beyond sensory cortices, it is unknown whether mice can perceive optogenetic manipulations from a wide range of brain regions besides their ascribed physiological function, and thus regardless of the perceptual experience it causes. To this aim, we systematically explored the mice’s ability to report various optogenetic perturbations from multiple brain regions to obtain rewards and avoid punishments. Specifically, we designed an optogenetic-cue alternation task, in which we asked mice to change head direction and return to lick a previously rewarded sipper to report if they detected any brain perturbation. Notably, none of the optogenetic manipulations assayed caused this return behavior initially. However, we found that mice could correctly report all optogenetic perturbations assayed after training. Other behavioral tasks confirmed this finding. In sum, we found that mice can detect and learn to use brain perturbations to obtain rewards, suggesting the brain is capable of “monitoring” its self-activity.

## Materials and Methods

We used adult male and female mice weighing 20–30 g at the beginning of the experiment. Different strains of mice were used in this study C57Bl6J [wild-type (WT) mice], B6.Cg-Tg(Thy1-COP4/eYFP)18Gfng/J (hereafter referred to as *Thy1-ChR2 mice*), B6.Cg-Tg(Slc32a1-COP4*H134R/eYFP)8Gfng/J (*VGAT-ChR2* mice), mice were purchased from The Jackson Laboratory (RRID: IMSR_JAX:007612 and RRID: IMSR_JAX:014548, respectively). The Thy1-ChR2 mice expressed the light-activated ion channel, channelrhodopsin-2 (ChR2), fused to an enhanced yellow fluorescent protein (eYFP) under the control of the mouse thymus cell antigen 1 (Thy1) promoter (Kumar et al., 2013). The VGAT-ChR2 mice expressed the ChR2 under the control of the vesicular GABA transporter (VGAT; Zhao et al., 2011). Furthermore, we employed the Vgat-ires-cre mice because they express cre-recombinase enzyme under the control of endogenous Vgat promoter (RRID:IMSR_JAX:016962; Vong et al., 2011). Finally, we used TH-Cre mouse, which expresses cre-recombinase under the control of endogenous tyrosine hydroxylase promoter. The TH-cre mice were kindly donated by Bermúdez-Rattoni from Instituto de Fisiología Celular, Universidad Nacional Autónoma de México.

Mice were individually housed in standard laboratory cages in a temperature-controlled (22 ± 1°C) room with a 12/12 h light/dark cycle (lights were on 7 A.M. and off at 7 P.M.). All procedures were approved by the CINVESTAV Institutional Animal Care and Use Committee. Unless otherwise stated, mice were given *ad libitum* access to water for 60 min after testing. Chow food (PicoLab Rodent Diet 20) was always available in their homecage. All experiments were performed in the light period from 9 A.M. to 4 P.M.

### Viral constructs

The Cre-inducible adeno-associated virus (AAV) were purchased from *addgene*. For ChR2-eYFP (AAV5-EfIa-DIO-hChR2(E123T/T159C)-EYFP, #35 509, at titer of 1 × 10^13^ vector genome/ml (vg/ml), ArchT-tdTomato (AAV5/FLEX-ArchT-tdTomato), #28305, at titer of 7.0 × 10^12^ vg/ml and eYFP-vector (AA5V-EfIa-DIO EYFP), #27056, at titer of 1.0 × 10^13^ vg/ml. Viruses were divided into aliquots and stored at −80°C before their use.

### Stereotaxic surgery

Mice were anesthetized with an intraperitoneal injection of ketamine/xylazine (100/8 mg/kg). Then mice were put in a stereotaxic apparatus where a midline sagittal scalp incision was made to expose the skull to insert two holding screws.

#### Viral infection

A microinjection needle (30-G) was connected to a 10-μl Hamilton syringe and filled with AAV. Vgat-ires-cre or TH-cre mice were microinjected with AAV (0.5 μl) at a rate of 0.2 μl/min. The injector was left in position for five additional minutes to allow complete diffusion. In the case of Vgat-ires-cre, the microinjection was performed unilaterally in the lateral hypothalamus [LH, from bregma (mm): AP −1.3, ML ±1.0, and from dura (mm): DV −5.5] for the expression of ChR2 (LH^ChR2^), ArchT (LH^ArchT^), or eYFP (LH^eYFP^), and then a zirconia ferrule of 1.25 mm in diameter with multimode optical fiber (200 μm, Thorlabs) was implanted in LH [from bregma (mm): AP −1.3, ML ±1.0 and from dura (mm): DV −5.3]. TH-Cre mice were microinjected bilaterally with ChR2 in VTA [TH-VTA mice; from bregma (mm): AP −3.0, ML ±0.6, and DV −4.8], and ferrules were implanted in VTA [from bregma (mm): AP −3.0, ML ±1.2, and DV −4.3, 10° angle], mice were allowed one month for recovery and obtain a stable expression of ChR2 or ArchT.

#### Fiber optics and optrode implantation

An unilateral zirconia ferrule (200-μm core diameter and 0.39 NA FT200UMT; Thorlabs) was implanted in WT, Thy1-ChR2, and VGAT-ChR2 mice. Thy1-ChR2 mice were implanted in prefrontal cortex [*PFC^Thy1^*, from bregma (mm): AP +1.94, ML ±0.3, and DV −2.8], or in nucleus accumbens [NAc^Thy1^, from bregma (mm): AP +1.2, ML ±1.0, and DV −5.2]. For VGAT-ChR2 mice, the optical fiber was implanted in PFC in the same coordinates as in the *PFC^Thy1^* (PFC^VGAT^) or thalamic reticular nucleus [TRN^VGAT^, from bregma (mm): AP −1.55, ML ±2.5, and DV −3.25]. WT mice were implanted in the PFC in the same coordinates as the *PFC^Thy1^* (PFC^WT^).

For the experiment of two brain regions perturbed in the same subject, the Thy1-ChR2 (*n* = 4) and VGAT-ChR2 (*n* = 4) mice were implanted unilaterally with fiber optics in NAc (similar to NAc^Thy1^) and lateral cerebellum using the following coordinates: from bregma (in mm): AP −5.80, ML ±2.25, and DV −2.05 (Pendharkar et al., 2021).

The optrode comprised a homemade array of 16 tungsten wires (35 μm) formvar insulated (California Fine Wire Company), surrounded in a circular configuration by a single optical fiber was implanted in PFC in Thy1-ChR2 (*n* = 3) or VGAT-ChR2 (*n* = 3) mice [from dura (in mm): AP +1.94, ML ±0.3 and DV −2.8]. Mice were allowed one week for recovery from surgery.

### Optogenetic parameters

Mice expressing ChR2 were stimulated with a diode-pumped solid-state system blue at 473 nm (OEM laser) or green at 532 nm for ArchT opsin (Laserglow Technologies). The light output intensity at the optical fiber patch cord for ChR2 was 3 mW for *PFC^Thy1^*, whereas 15 mW for the remaining brain regions, while for ArchT stimulation, it was 20 mW. In control *PFC^WT^* mice, they were photo-stimulated at 3 mW in the optogenetic-cue alternation task and 15 mW for the frequency discrimination task. The light intensity was measured with an optical power meter with an internal sensor (PM20A, Thorlabs). The pulse lengths were 30 ms for ChR2 and continuous pulse for ArchT and were controlled by Med Associates Inc., software, and TTL signal generator (Med Associates Inc.).

### Extracellular optrode recordings

Thy1-PFC and VGAT-PFC mice with optrode implant were recorded for 20 min in a scanner laser frequency task (Prado et al., 2016), where they received a random stimulation frequency during 1 s “on,” followed by 2 s “off,” and the subsequent frequency was randomly chosen. The stimulation frequencies were Control (0 Hz), 4, 7, 10, 14, and 20 Hz. Mice were recorded in a maximum of seven consecutive sessions.

Neural activity was recorded using a Multichannel Acquisition Processor system (Plexon) interfaced with a Med Associates conditioning side to record behavioral events simultaneously. Extracellular voltage signals were first amplified 1× by an analog headstage (Plexon HST/16o25-GEN2-18P-2GP-G1), then amplified (1000×) sampled at 40 kHz. Raw signals were bandpass filtered from 154 Hz to 8.8 kHz and digitalized at 12-bit resolution. Only single neurons with action potentials with a signal-to-noise ratio of 3:1 were analyzed. The action potentials were isolated online using voltage-time threshold windows and three principal components contour templates algorithm. A cluster of waveforms was assigned to a single unit if two criteria were met: interspike intervals were larger than the refractory period set to 1 ms, and if a visible ellipsoid cloud composed of the 3-D projections of the first three principal component analysis of spike waveform shapes was formed. Spikes were sorted using Offline Sorter software (Plexon). Only time stamps from offline-sorted waveforms were analyzed. All electrophysiological data were analyzed using MATLAB (The MathWorks Inc.; Fonseca et al., 2018).

## Behavioral methods

### Habituation phase: training to alternate between sippers

Mice were initially trained in a behavioral box (31 × 41.5 cm) equipped with two sippers at each side of the frontal wall of the box. Each sipper was calibrated to deliver two drops of sucrose 10% (3–4 μl each drop) or two air-puffs (10 psi) via a computer-controlled solenoid valve (Parker). The two sippers were semi-divided by a central acrylic wall covering 2/3 parts. This forced mice to cross between sippers by the remaining 1/3 part of the wall that remains open, facilitating the detection of mice’s crosses using a photobeam in the central wall that was recorded when mice were halfway between the two sippers.

Mice were water-deprived for 23 h and then placed in the behavioral box. In the first phase, they had to learn to alternate between the two sippers. In each trial, mice had to lick an empty sipper twice to receive two drops of sucrose in the third and fourth lick. After that, they need to move to the opposite sipper to start a new trial and obtain more sucrose. Each session lasted 30 min daily. To consider that a mouse learned, they had to complete 60 trials in one session.

### Optogenetic-cue alternation task

Once mice learned to alternate between sippers, they were placed in a similar task, but this time in 50% of trials, a cue was delivered pseudorandomly. This consisted of a tone 2 kHz + laser at 20 Hz (or continuous pulse for ArchT) delivered when mice were heading halfway toward the opposite sipper; at this moment, the mice had to return to the previous trial’s sipper to initiate a new rewarded trial. If they licked on the opposite sipper, the mice were punished with two air puffs delivered in the third and fourth lick. The mice were trained on this task until they reached five consecutive sessions with >50% of punishments avoided. All sessions were 30 min long.

A new group of naive the Thy1-ChR2 and VGAT-ChR2 mice were implanted with an optical fiber in the NAc and one in the lateral cerebellum from the same hemisphere to stimulate two brain regions. First, these mice received stimulation only in the NAc as a cue until they reached >50% correct cue trials for at least three consecutive sessions in the optogenetic-cue alternation task. Then the stimulation was switched to the lateral cerebellum until they achieved the learning criteria again. After that, some mice were exposed to a fake laser session to finally test them in the task variant with interleaved stimulations from both brain regions randomly in the last three sessions.

### Frequencies and pulse task variants

The same mice were also tested in five consecutive sessions in the optogenetic-cue alternation task. The laser frequency changed trial by trial in random order at 4, 7, 10, 14, and 20 Hz in 50% of total trials. In the subsequent five sessions, the laser frequency was fixed constant (20 Hz), but the number of pulses varied (1, 3, 6, 11, 16, and 20 pulses) in 60% of total trials. Some mice started with variable pulse variants to counterbalance between subjects, and others began with the laser frequency variant. In all experiments, the task lasted 30 min daily.

### Frequency discrimination task

Mice were placed in an operant chamber (Med Associates Inc.) equipped with three ports on one wall. The central port had a head entry detector (infrared sensor) and two lateral ports, and each contained a sipper that could deliver 2-μl dropper lick of 10% sucrose as a reward or a 10-psi air-puffs as a punishment. The task lasted for 30 min and consisted of delivering a laser stimulation after each head entry in the central port; the laser frequencies were either 10 or 20 Hz (1 s). Then, mice had to lick in the corresponding lateral port to receive two drops of sucrose. If mice licked in the incorrect port, they received two air puffs. A clicker sound marked the start and the end of the trial. A correct trial was when the mice licked in the port associated with the stimulation frequency. Mice learned the task when they reached 85% of correct trials in three consecutive sessions. In a fake laser session, mice were connected to inactive optical fiber attached to the active fiber.

### Frequency categorization task

Mice were trained to discriminate between 10- and 20-Hz frequencies and then underwent a categorization task to indicate whether they received a lower or higher frequency. This task variant lasted 30 min, and it was similar to the frequency discrimination task, but one port was associated with lower frequencies (10, 12, and 14 Hz), whereas the opposite port corresponded to higher frequencies (16, 18, and 20 Hz).

### Lever-press self-stimulation task

Well-fed (sated) mice were placed in an operant chamber with two levers located in opposite walls (Med Associates Inc.). One lever was associated with the delivery of 1 s of optogenetic stimulation (20 Hz), whereas the other was inactive (30 min daily sessions). After pressing the active lever, mice received a train of optogenetic stimulation (1 s “on” + 2 s of time out). After three sessions, the levers were switched, and thus the previously active lever was now inactive and vice versa. Mice were tested during four more sessions. In the extinction phase, the last three sessions, both levers were inactive.

### Open field center self-stimulation

Sated animals were placed in a circular open field (diameter 45 cm) with a central circular flat glass (diameter 10 cm, thick 1.83 mm). In this task, mice had to cross to the central circular glass to receive optogenetic stimulation (1 s, 20 Hz) plus 2 s of time out for three sessions (30 min each). In the last four sessions, optogenetic stimulation was not delivered as an extinction test.

### Real-time conditioned place preference (rtCPP)

Well-fed mice were placed in a rectangular acrylic arena (20 × 20 × 42 cm) divided in half by two different visual cues. One side had black/white stripes, and the other side had black/white circles. Mice were allowed to cross between sides freely. On day 1, mice explored the arena for 10 min (pre-test). On the following days (sessions 2–4), the 20-min consecutive sessions, they were conditioned on the side where they spent less time (or in the more preferred for LH^ArchT^ mice) by pairing optogenetic stimulation (20 Hz, 1 s “on” – 2 s “off” for ChR2 and eYFP, or continuous pulse 1 s “on” + 2 s “off” for ArchT) each time they crossed and stayed in the less preferred side. On day 5, mice were placed in the arena again for 10 min without laser stimulation (test day). The preference index in the conditioned and unconditioned sides was computed by dividing the time spent on each side by the total exploration time (Gil-Lievana et al., 2020).

### Open-loop task

Water-deprived LH^ArchT^ and LH^eYFP^ mice were placed in an operant chamber (Med Associates Inc.), with a central sipper port to dispense 2-μl drops of sucrose 10% at each lick. They were stimulated in blocks of 1 min “laser on” – 1 min “laser off” during 30. All licking responses were recorded by a contact lickometer (Med Associates Inc.).

### Closed-loop task

Well-fed LH^ChR2^ and LH^eYFP^ control mice were placed in an operant chamber (Med Associates Inc.), equipped with a central sipper port to dispense drops sucrose 10% each lick and a head entry detector (infrared sensor; Med Associates Inc.). Each head entry triggered a train of optogenetic stimulation during 1 s (20 Hz) + 2 s time out in 30 min. Licks and head entries were recorded. Mice were placed in this task in three sessions, whereas the subsequent three were for extinction (without laser).

### Histology

After experiments were finished, mice were treated with an overdose of pentobarbital sodium (0.1 ml 10 mg/kg), and they were transcardially perfused with PBS followed by 4% paraformaldehyde (PFA). Brains were removed, stored for 1 d in PFA 4%, and later exchanged with a 30% sucrose/PBS solution. Brains were sectioned at 40-μm coronal slices. Slices were placed in a mounting medium (Dako) to visualize the fluorescence of the reporter (eYFP or tdTomato) and implantation sites. Images were taken with a Nikon eclipse e200 and with a progress gryphax microscope camera, using a 4× objective, and for visualization purposes, the image contrast was improved with Adobe Photoshop CS5.1 software.

### Quantification and statistical analysis

Data were analyzed in MATLAB R2021a (The MathWorks Inc.) and GraphPad Prism. Unless otherwise mentioned, data were expressed as a mean ± SEM, and statistical analysis was performed from a Student’s *t* test and two-way ANOVA test followed by a Holm–Sidak or Dunnett *post hoc* and repeated-measures ANOVA in case of comparison across sessions.

The firing rate was compared for electrophysiological recordings by a nonparametric Kruskal–Wallis test followed by a Tukey–Kramer *post hoc*. Neurons whose firing rates during the laser “on” period were significantly different (*p *<* *0.05) at any laser frequency relative to the activity in the control trials were considered as modulated. The difference between firing rates during laser stimulation versus control trials determined the modulation sign (i.e., increased or decreased).

For the synchronicity index, the fraction of co-fired neurons was calculated in each trial (from −0.5 to 2 s aligned to laser onset time = 0 s), within a 10-ms bin resolution. We counted (C) when a neuron fired (C = 1) or C = 0 if the cell did not fire in each bin. Then, we divided each trial bin by the number of neurons recorded simultaneously per session (separated for each laser frequency), and then we averaged all subjects and sessions.

## Results

### Optogenetic brain perturbations transiently alter spiking homeostasis and increase synchronous firing

**Figure 1.**
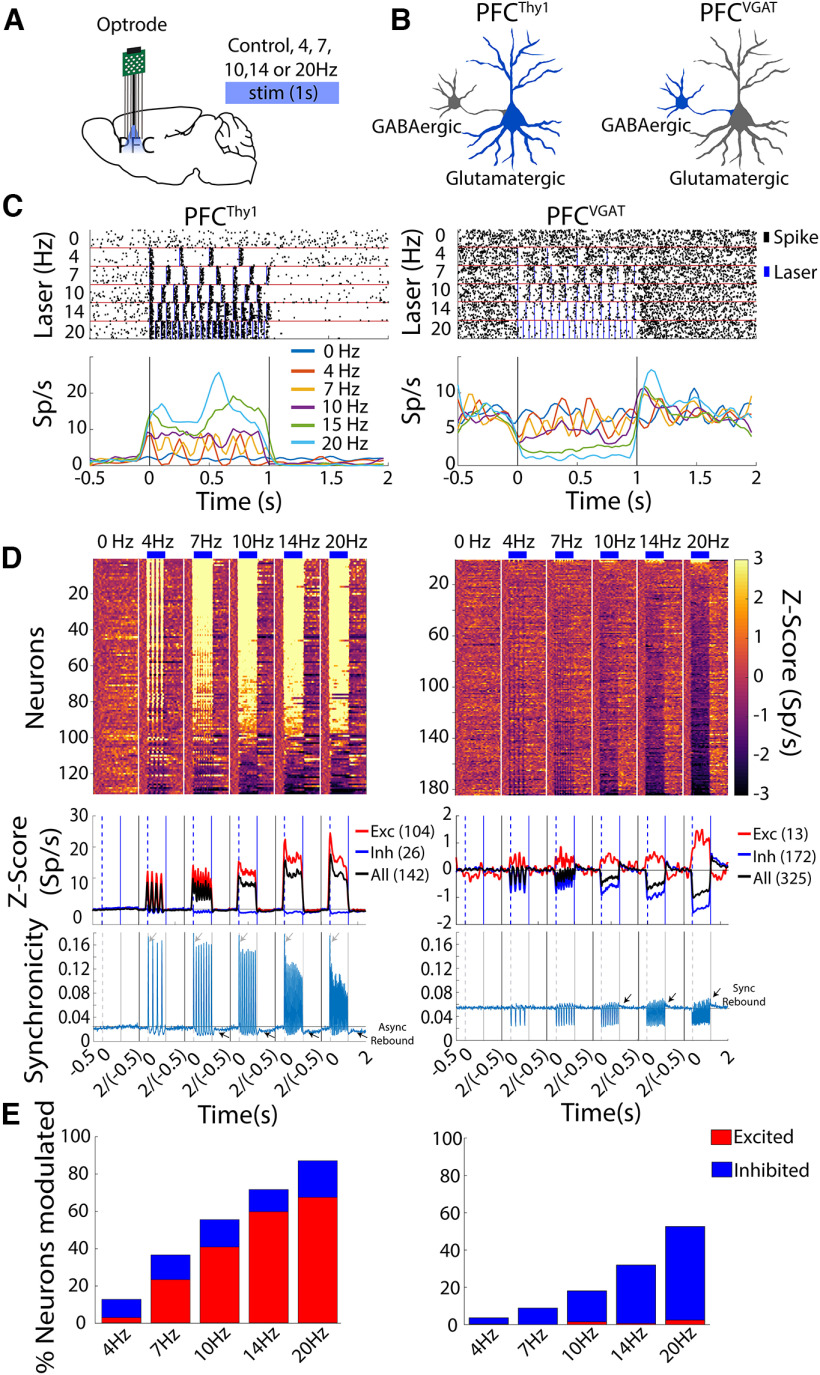
Optogenetic stimulation of PFC^Thy1^ or PFC^VGAT^ transiently impacts E/I activity balance, evoking opposite neuronal responses in a laser frequency-dependent manner. ***A***, Schematic representation of PFC recording sites in mice with optrodes record and stimulation at different frequencies. ***B***, Diagram of neurons expressing ChR2 that were optogenetically stimulated (in blue). In PFC^Thy1^ mice, the stimulation drives the activation of glutamatergic neurons, whereas, in PFC^VGAT^ mice, it activates cortical GABAergic neurons inducing an indirect inhibition of glutamatergic neurons. ***C***, Representative examples of two neurons modulated. Upper panel, raster plot of one neuron recorded from PFC^Thy1^ (left) and the other in PFC^VGAT^ (right) aligned to laser onset (time = 0 s). Below are shown the PSTHs, respectively. Vertical lines indicate laser onset. ***D***, Population activity. Upper panel, Heat map of neuronal population activity from PFC^Thy1^ (left) or PFC^VGAT^ (right), normalized to Z-scores, vertical white lines by laser frequency. Nonmodulated neurons are not plotted. Bottom panel, Population PSTH activity. Dashed lines indicate laser onset, blue line laser offset, and the baseline (−0.5 to 0 s) black line. Below is the synchronicity index, which reflects the fraction of simultaneously recorded neurons that co-fire on a trial-by-trial basis within a 10-ms bin resolution around laser onset. Qualitatively similar results are found at 1-ms resolution (data not shown). Dashed lines indicate laser onset, solid gray line laser offset, and the baseline is shown in the black line. ***E***, Percentage of neurons modulated by different laser frequencies for PFC^Thy1^ (total recorded neurons, *n* = 142, left) or PFC^VGAT^ (total neurons, *n* = 325, right).

We first characterize whether optogenetic perturbations transiently affect the spiking homeostasis [i.e., the excitatory/inhibitory (E/I) balance; Maffei and Fontanini, 2009; Dehghani et al., 2016; Prado et al., 2016; Dalgleish et al., 2020; Berntson and Khalsa, 2021]. To do this, from freely moving Thy1-ChR2 or VGAT-ChR2 mice, we performed optrode recording in the prefrontal cortex (PFC). We choose PFC because Thy1-ChR2 mice constitutively express the ChR2 in pyramidal layer V glutamatergic neurons (Arenkiel et al., 2007; Kumar et al., 2013), while VGAT-ChR2 mice constitutively express ChR2 in GABAergic^VGAT+^ neurons, respectively (Zhao et al., 2011; Babl et al., 2019). Thus, in VGAT-ChR2 mice, when GABAergic neurons are optogenetically activated, they produce an indirect but extensive inhibition, probably reaching off-target brain regions (Babl et al., 2019). We hypothesized that optogenetic manipulation of either glutamatergic or GABAergic neurons in PFC briefly perturbed spiking homeostasis, albeit with opposite modulatory sign. Mice were optogenetically stimulated in a frequency scanner test, while optrode recordings were simultaneously performed (Prado et al., 2016). We found that optogenetic activation of cortical glutamatergic or GABAergic neurons produced phase-locked firing and an opposite modulatory activity as the laser frequency increased (Fig. 1*A–D*; Prado et al., 2016). A transient E/I imbalance caused by optogenetic perturbations was also observed in the overall population activity of all neurons recorded (Fig. 1*D*, see black traces). In addition, the optogenetic stimulation in Thy1-ChR2 mice caused robust synchronicity, the fraction of co-fired neurons. The synchronicity was maximal at all laser frequencies tested in the first laser pulse (Fig. 1*D*, see gray arrows), followed by a gradual decrease for subsequent laser pulses. Unexpectedly, after activating cortical glutamatergic neurons, we observed an epoch of asynchronous rebound activity that was stronger as the frequency increased (see Fig. 1*D*, black arrows async rebound). In contrast, the firing rate of VGAT-ChR2 mice displayed a brief inhibition during each laser pulse and rebound synchronicity at the offset of each laser pulse that gradually increased as the laser frequency increased (Fig. 1*D*, right panel, see arrows rebound sync). Thus, optogenetic activation of both glutamatergic and GABAergic neurons produced an unbalanced E/I activity and a spike synchronization.

Briefly, in the Thy1-ChR2 mice, 67% of PFC neurons increased their excitability, and 20% inhibited after stimulation at 20 Hz. In contrast, in the VGAT-ChR2 mice, 3% of neurons were excited, and 50% were inhibited (Fig. 1*E*). The optogenetic-induced E/I imbalance might arise as a side effect of the most attractive feature of optogenetics, which is the ability to drive synchronous action potentials of a single cell type (Dehghani et al., 2016; Prado et al., 2016; Dalgleish et al., 2020). As a result, ChR2 expressing cells will exhibit phase-locked firing entrained to laser and increased synchronicity (Prado et al., 2016; Gill et al., 2020). Perhaps, the spiking homeostasis is triggered as a compensatory mechanism to recover the E/I balance.

### Mice can perceive and actively report optogenetic perturbations

**Figure 2.**
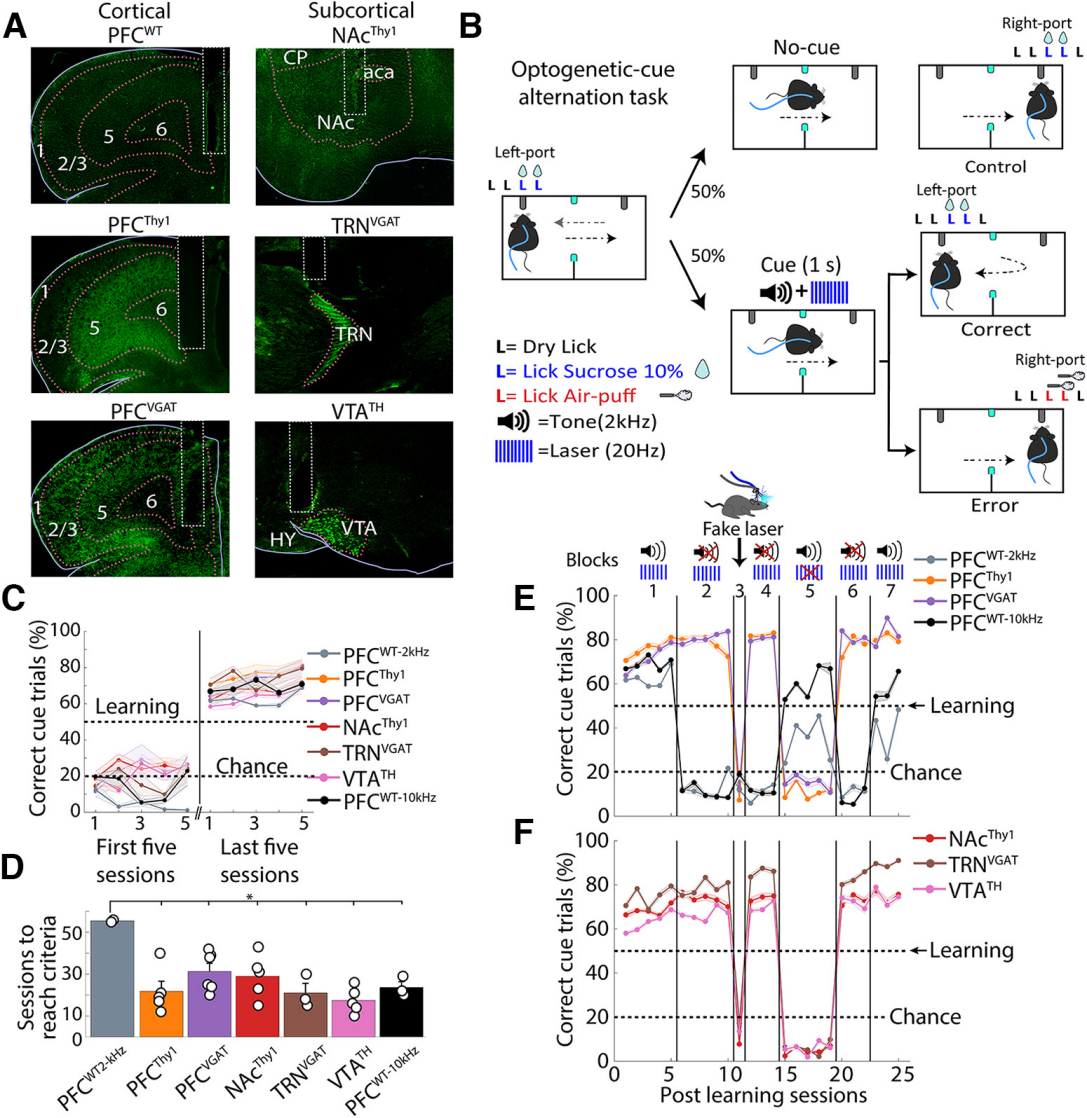
Mice learn to use optogenetic manipulations as a cue regardless of the perturbed cell type or brain region. ***A***, Representative images for fiber optics implantation and stimulation sites. Left pictures show unilateral optical fiber implanted in prefrontal cortices in WT (PFC^WT^), transgenic Thy1-ChR2 (PFC^Thy1^), and VGAT-ChR2 (PFC^VGAT^) mice. Right pictures show fiber optics in subcortical regions, including the NAc in Thy1-ChR2 (NAc^Thy1^), the TRN in VGAT-ChR2 (TRN^VGAT^), and the ventral tegmental area in TH-Cre (VTA^TH^) mice. ***B***, Schematic of the optogenetic-cue alternation task, where mice had to alternate between two sippers to receive two drops of 10% sucrose from each one. When the mice broke the photobeam, located halfway between the two sippers (cyan squares), a cue [tone (2 kHz) + laser (20 Hz), 1 s] was randomly delivered. The cue instructed them to return to the previously rewarded port to be rewarded again and avoid punishment. A dry lick is a lick given to the empty sipper. ***C***, Task performance in the initial and last five training sessions. Learning criteria (horizontal dashed line at 50%) were reached when mice avoided punishment in >50% of cue trials in five consecutive sessions. Correct trials separated as Hit and Correct Rejections are shown in Extended Data Figure 2-1, where only Hits increased their proportion when mice learned the task. Note that PFC^WT-10kHz^ mice were trained with a more easily perceived auditory tone 10 kHz, than a 2-kHz tone that was barely perceptible to mice, as shown in Extended Data Figure 2-2. Error bars indicate SEM. ***D***, Sessions to reach the learning criteria. ***E***, Task performance postlearning in mice with PFC optogenetic stimulation (block 1). In block 2, the tone was removed. In block 3, mice were tested with a “fake laser.” After reacquisition (block 4 laser only), block 5 began, where the tone was the cue only. Then in block 6, we re-tested laser only condition. Finally, we repeated the laser+tone condition in block 7. ***F***, Similar to panel ***E***, except that stimulation was delivered in subcortical structures (NAc^Thy1^, TRN^VGAT^, and VTA^TH^); *p <* *0.01 ANOVA Dunnett *post hoc* relative to PFC^WT^ control.

We hypothesized that regardless of cell type or brain region perturbated, the mice could perceive optogenetic stimulations and learn to use it as a conditioned cue to solve a task. To test this idea, we implanted optical fibers in the PFC and the nucleus accumbens (NAc), as we described in our previous work (Prado et al., 2016). For the PFC, we tested glutamatergic neurons in Thy1-ChR2 mice (PFC^Thy1^; Porrero et al., 2010), or GABAergic neurons in VGAT-ChR2 mice (PFC^VGAT^; Zhao et al., 2011). For NAc, in Thy1-ChR2 mice, we stimulated glutamatergic afferent fibers. We also included neuromodulatory dopaminergic neurons in the ventral tegmental area (VTA^TH^, ChR2 expression in TH-Cre mice; Gil-Lievana et al., 2020). Furthermore, we implanted an optical fiber in the thalamic reticular nucleus (TRN), a structure related to “awareness” that highly express ChR2 in the VGAT-ChR2 mice (TRN^VGAT^; Zhao et al., 2011; Wimmer et al., 2015). WT mice with an optical fiber in PFC served as a control (PFC^WT^; Fig. 2*A*).

10.1523/ENEURO.0216-22.2022.f2-1 Extended Data Figure 2-1 Mice learn to return to the previously rewarded port only when the optogenetic cue is present (Hit trials). ***A***, Table of different types of trials Correct Rejection, False Alarm, Hits, and Misses. In our task, mice had to continue alternating between sippers in no-cue trials (Correct Rejections), and very few False Alarm responses were observed. In contrast, in cue trials, lick responses were given in the previously rewarded sipper (Hits), and a few Misses were made. ***B***, Schematic of the optogenetic-cue sipper alternation task separated by trial type. ***C***, Task performance in the first five and last five sessions during the task. After reaching the learning criteria (horizontal dashed line), only hit trials increased while misses decreased. Correct rejection and False alarm maintained the same task performance as in the first five sessions. Error bars indicate SEM. Download Figure 2-1, TIF file.

10.1523/ENEURO.0216-22.2022.f2-2 Extended Data Figure 2-2 The 2-kHz tone was less efficient than the optogenetic stimulation as a cue. ***A***, Correct trials of PFC^WT^ mice that did not reach the learning criteria trained at 2 kHz in the Optogenetic-cue sipper alternation task. Individual mice are shown in gray, and the mean ± SEM is shown in purple. Black dots showed the last session of each subject. ***B***, The tone frequency was changed to 10 kHz. The task performance of the three nonlearners subjects, from panel ***A***. The same subjects were also trained with a 10-kHz tone. Note that after the tone was changed from 2 to 10 kHz, they rapidly learned the task (gray dash line learning criteria). ***C***, Sensitivity index (d prime, d’) was computed for the first and last five sessions. Transgenic mice showed values above d’ > 1. In contrast, PFC^WT-2KHz^ exhibited values below d’ < 1, indicating that although they reached the learning criteria, they could not detect the cue as efficiently as transgenic mice or PFC^WT-10kHz^. PFC^WT Non-L^ refers to nonlearners. ***D***, Total trials (Cue + No-Cue). Each dot represents an individual subject; *p <* *0.05 paired *t* test. Download Figure 2-2, TIF file.

To demonstrate that optogenetic perturbations can serve as a discriminative stimulus, water-deprived mice were initially habituated to alternate between two sipper tubes to obtain two drops of sucrose from each (data not shown). After that, they were trained in an optogenetic-cue alternation task, in which 50% of trials (no-cue), mice had to alternate between two sippers to receive sucrose (Fig. 2*B*). In the other 50% of trials (cue), mice received a combined cue (tone 2 kHz at 80 dB + laser 20 Hz, 1 s) halfway between sipper tubes. The combined cue instructed them to return to the previously rewarded port to be rewarded again and avoid punishment (correct cue-trial). If mice ignored the cue, i.e., they did not change direction and lick the opposite sipper, two air puffs were delivered (Fig. 2*B*, error trial; Movie 1). We judged that the mice learned the task if, in five consecutive sessions, >50% of cue trials were correct (Fig. 2*C*; Extended Data Fig. 2-1). A 2 kHz at 80-dB auditory tone was chosen because it is barely perceptible to mice (Heffner and Heffner, 2007), resulting in a greater saliency toward the optogenetic cue. Indeed, of the 10 PFC^WT-2kHz^ mice tested, only two solved the task, but they took significantly more sessions to learn than transgenic mice (Fig. 2*D*). The remaining eight WT mice never reached the learning criteria (PFC^WT non-L^ trained with a tone 2 kHz + laser); one mouse was even tested for 130 sessions (Extended Data Fig. 2-2*A*). We then re-used three nonlearning mice, but now we trained them with a higher tone frequency of 10 kHz (which they readily perceive). These three control mice rapidly reached the learning criterion (Fig. 2*C*,*D*; Extended Data Fig. 2-2*B*, PFC^WT-10kHz^). Importantly, we found that all transgenic mice trained with tone 2 kHz + laser rapidly acquired the task (Fig. 2*C*,*D,E*, cortical brain structures, *F*, subcortical brain structures, see block 1), suggesting that they could perceive optogenetic brain perturbations.

Movie 1. Optogenetic-cue alternation task, tone + laser. Example of transgenic mice in block 1. 10.1523/ENEURO.0216-22.2022.video.1

We then showed that transgenic mice used only brain perturbations to guide behavior. To do this, the 2-kHz tone was removed from the combined cue, and mice received the laser only (Fig. 2*E*,*F*, block 2, laser only). We observed that the transgenic mice maintained their task performance above learning criteria, even when the laser was the only feedback cue (Fig. 2*E*,*F*, see block 2; Movie 2). PFC^WT^ control mice trained with either 2- or 10-kHz tone dropped their task performance at the chance level after the tone was removed, demonstrating that the auditory tone guided WT mice’s behavior. This result also suggests that under our experimental conditions, WT mice did not rely on sensory information evoked by the blue light laser or its thermal effects for discrimination per se (Owen et al., 2019). Unlike controls, all transgenic mice rapidly acquired the task, suggesting they used optogenetic perturbations as a discriminative stimulus.

Movie 2. Laser only session. Transgenic mice in block 2, without tone. 10.1523/ENEURO.0216-22.2022.video.2

To determine whether transgenic mice use the blue light per se as a cue (Danskin et al., 2015), a “fake laser” was used to study this in more detail. In this condition, mice could see the blue laser outside the skull without receiving optogenetic stimulation (Movie 3). It was found that the task performance of all transgenic mice dropped at the chance level (Fig. 2*E*,*F*, block 3, fake laser), demonstrating that transgenic mice did not use light as a discriminative stimulus. We tested the laser only once again to recover transgenic mice performance (block 4, laser only). Then we inquired whether the tone served as a discriminative stimulus. Thus, the laser was replaced for the tone as a cue (Fig. 2*E*,*F*, block 5, tone only). Unlike the WT mice, which increased task performance with the tone only, the transgenic mice exhibited a drastic drop in task performance (Movie 4). In block 6 (laser only), we recovered transgenic mice’s task performance. Finally, given that in block 5 (tone only) and last block 7 (tone+laser), the WT mice exhibited a lower task performance than in the initial block 1 (tone+laser), it suggests that WT mice exhibited a slight extinction induced by the extensive testing with the laser only condition (i.e., blocks 2 and 4). All in all, our results demonstrate that transgenic mice neglected the tone and used optogenetic perturbations to solve the task, perhaps because neuronal perturbations are a more salient source of information to guide behavior.

Movie 3. Fake laser session. Transgenic mice in block 3, without tone and with the flashing light from the laser. 10.1523/ENEURO.0216-22.2022.video.3

Movie 4. Tone only session. Transgenic mice without laser and with a tone as a cue. 10.1523/ENEURO.0216-22.2022.video.4

### Mice can learn optoception even when the optogenetic perturbation is the only cue

**Figure 3.**
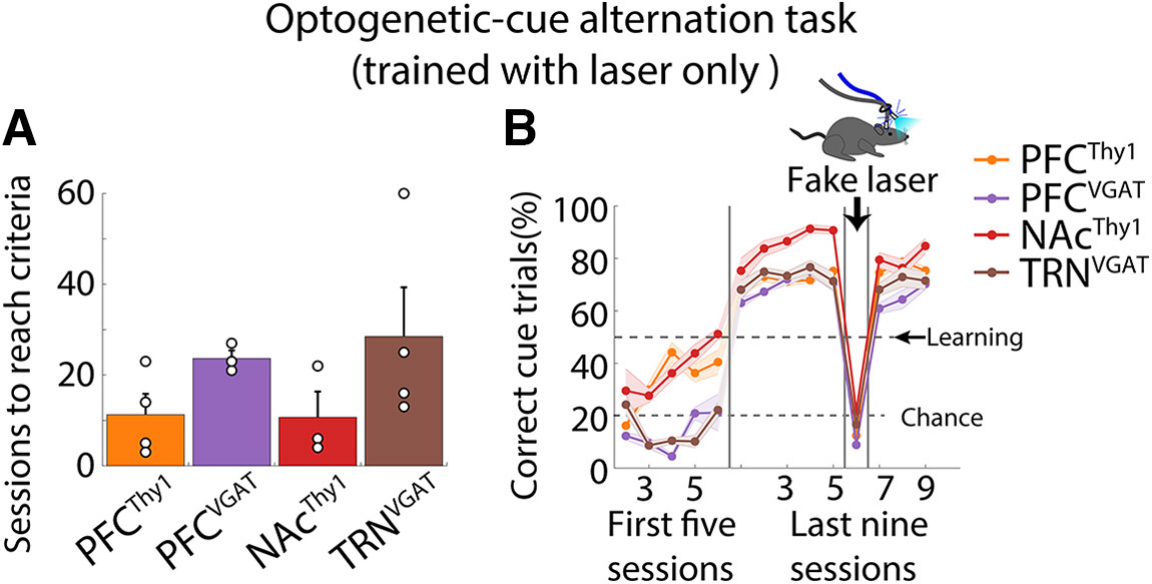
Optoception was induced by optogenetic stimulation only. ***A***, The number of sessions needed to reach the learning criteria. Mice were trained in optogenetic-cue sipper alternation tasks, as shown in Figure 1*B*, but with the laser only as a cue). Each dot represents an individual subject. ***B***, Percent of correct cue trials. Note the drop in task performance during the “fake laser” session, demonstrating that mice used the interoceptive (or any sensory-motor) effects induced by the optogenetic stimulation only as a conditioned cue. Error bars indicate SEM.

Then, and perhaps most importantly, we demonstrate that transgenic mice do not need an exteroceptive tone to learn. For this, we trained naive mice but this time only using the laser only as a cue. All transgenic mice assayed acquired the task, even when only the optogenetic stimulation served as a discriminative cue (Fig. 3). These data demonstrate that mice learn to use optogenetic brain perturbations as a perceptible cue to guide behavior.

### Mice can even perceive one single laser pulse

**Figure 4.**
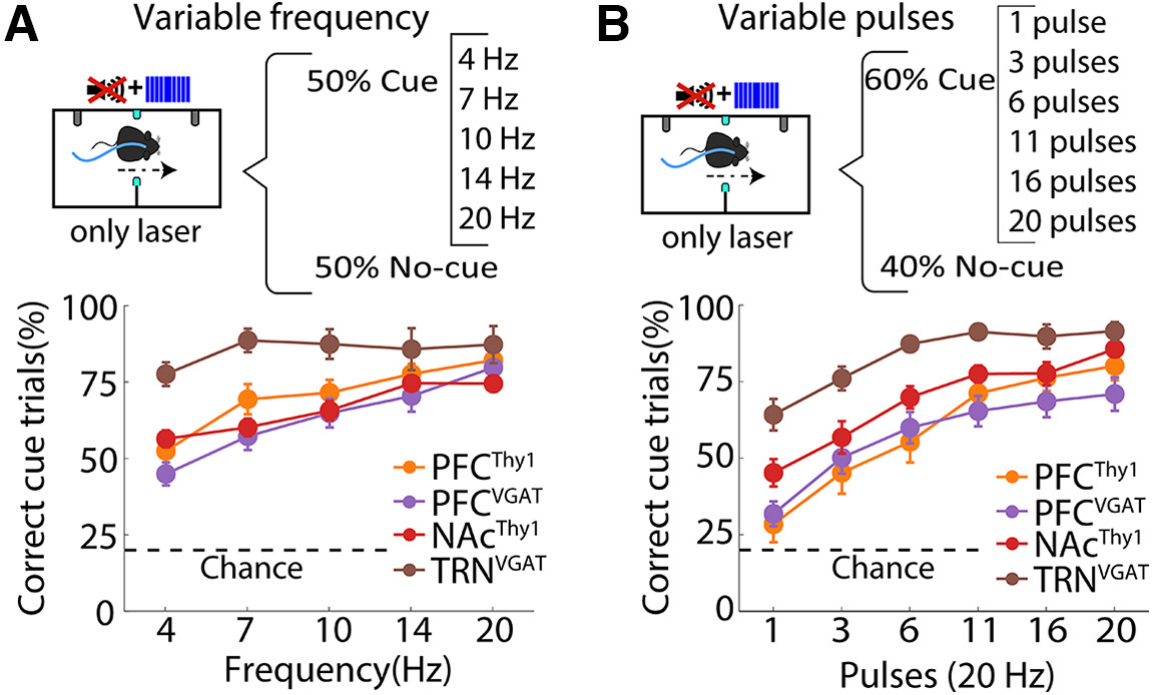
Mice can use optogenetic stimulation only as a cue and generalize to other laser parameters. ***A***, Upper panel, Schematics of the modified optogenetic-cue alternation task protocol where one out of five frequencies were randomly delivered in 50% of the trials. Bottom panel, Correct cue trials (correct frequency trials/total frequency trials). WT mice were not tested in these task variants because they did not perceive the laser only (Fig. 1*E*). ***B***, Upper panel, Structure of the modified pulse task variant. In this variant, one out of six laser pulses (from 1 to 20) were randomly delivered in 60% of the trials. Below are the correct cue trials (correct pulse trials/total pulse trials). Note that TRN^VGAT^ mice were more proficient in both task variants.

Having demonstrated that mice could use optogenetic stimulation as a cue and since optrode recordings unveiled that even a single pulse and ≥ 4 Hz laser frequencies could induce a robust phase-locked firing in PFC^Thy1^ and PFC^VGAT^ mice (Fig. 1). We then characterized the importance of the stimulation parameters to experience optoception, namely frequency and number of pulses. Thus, the same mice in Figure 2 were trained in two variants of the optogenetic-cue alternation task. Consequently, the laser frequency was randomly varied on a trial-by-trial basis (4–20 Hz). The correct responses gradually increased as the frequency reached 20 Hz (Fig. 4*A*). In the second task variant, the number of pulses was changed (fixed at 20 Hz frequency). We found a gradual increase in task performance when the pulses increased (from 1 to 20). In some cases, mice could even detect a single pulse, as happened in both NAc (one-sample *t* test; *t*_(4)_ = 2.76; *p* < 0.05 relative to chance level) and TRN (one-sample *t* test; *t*_(2)_ = 4.45; *p* < 0.05; Fig. 4*B*). All regions stimulated in these tasks’ variants showed a similar detection profile, except TRN^VGAT^ mice, which were more sensitive and outperformed in both task variants the other groups. Our results align with previous observations, suggesting that perceptual decisions can be driven by highly sparse neuronal activations (Houweling and Brecht, 2008; Huber et al., 2008; Dalgleish et al., 2020; Gill et al., 2020). It was concluded that mice could also discriminate between different sensations elicited by distinct optogenetic parameters.

### Mice learned two sets of instructions from two different laser frequencies

**Figure 5.**
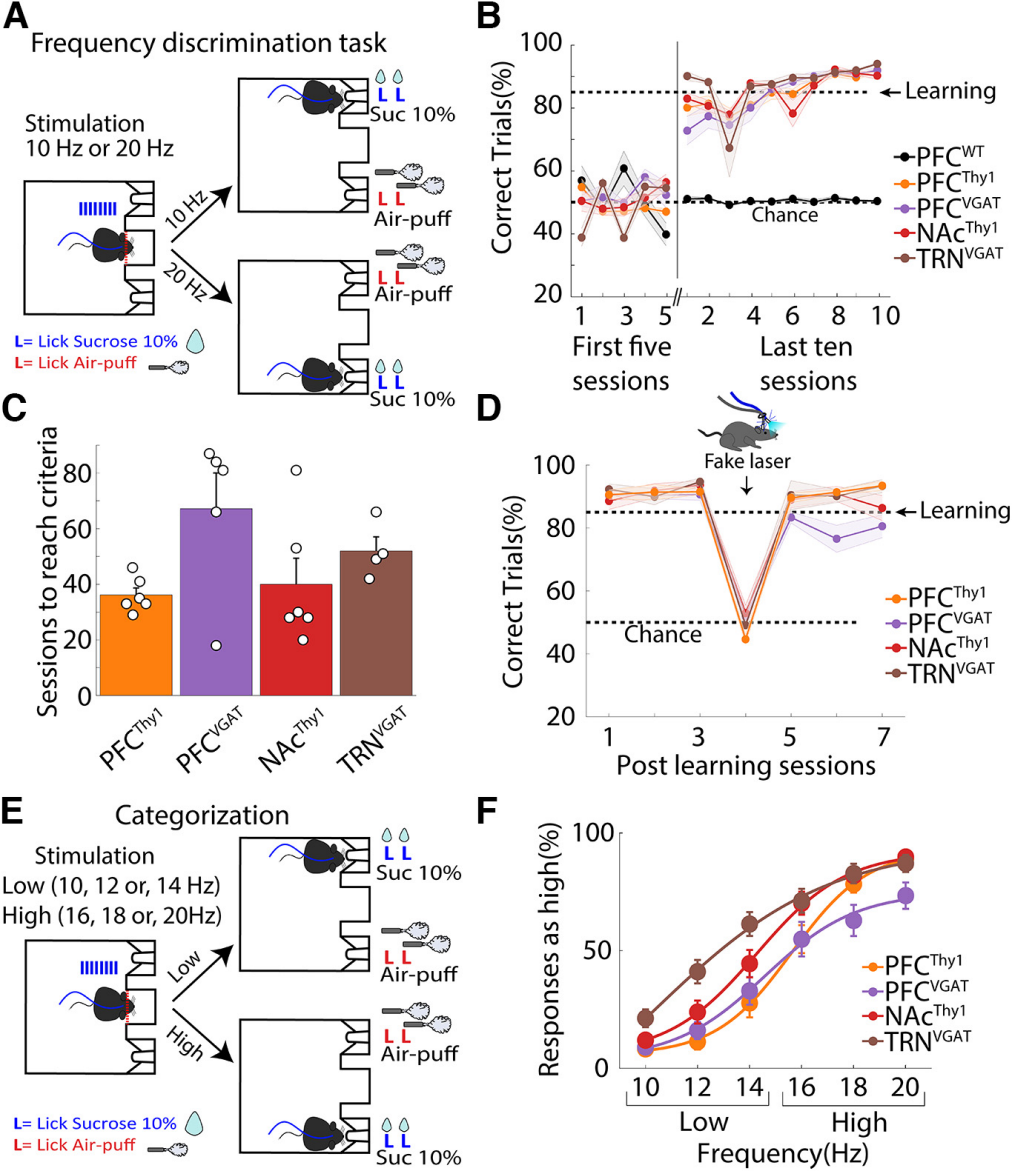
Mice use different laser frequencies to distinguish two actions. ***A***, Scheme of the frequency discrimination task. In this task, on head entry in the central port (red dashed line), the laser was turned “on” 1 s at 10 or 20 Hz, whereupon mice were required to lick in the lateral ports to receive either two drops of sucrose as a reward or two air-puffs as punishment (lateral ports were counterbalanced). ***B***, Correct trials were plotted for the initial five sessions and the last ten sessions after subjects reached the learning criteria (85% correct trials in 3 consecutive sessions). The control PFC^WT^ mice could not learn even after 90 training sessions. Error bars indicate SEM. ***C***, The time needed to reach the learning criteria. ***D***, Task performance in subjects that learned the task before and after testing with a “fake laser” in which mice could see the blue light outside the skull but did not receive any optogenetic stimulation. ***E***, Structure of the generalization task, mice had to categorize 10-, 12-, and 14-Hz frequencies as “low” and 16-, 18-, and 20-Hz frequencies as “high” by licking the lateral ports. ***F***, Psychometric function for choosing the “high” port. As the laser frequency increases, mice prefer the “high” port more, confirming that they categorized the different laser frequencies. This procedure was counterbalanced across mice.

We explored whether mice could learn two different sets of instructions based on the laser frequency delivered to the same brain area. In terms of classical perceptual studies, these instruction sets would correspond to two different task paradigms. First, mice were trained in a laser frequency discrimination task (Fig. 5*A*), where after visiting the central port, they received either a 10 or 20 Hz stimulation whereupon they had to lick in one of the two lateral ports; one frequency signaled the delivery of sucrose in left port and the other that sucrose is in the right port. If they chose the opposite port, they were punished with two air puffs (Movie 5). The control PFC^WT^ mice could not learn the task even after 90 training sessions (Fig. 5*B*), demonstrating that they did not use the temperature rise elicited by 10- and 20-Hz 1-s blue laser stimulation as a discriminative cue (Owen et al., 2019). In contrast, transgenic mice learned this task regardless of the cell type (glutamatergic and GABAergic) and brain region stimulated, the PFC, NAc, and TRN (Fig. 5*B*). All groups learned in a similar number of sessions (one-way ANOVA; *F*_(3,17)_ = 2.76, *p *=* *0.074). Although the PFC^VGAT^ group exhibited a nonsignificant trend in requiring additional sessions to reach learning criteria (Holm–Sidak’s multiple comparisons test, *p*s > 0.05; Fig. 5*C*). These experiments show that transgenic mice can discriminate between different optogenetic stimulation frequencies (Fridman et al., 2010). In the fake laser session, mice did not use the different visible light intensity generated from the laser since their task performance was at chance level that for this task was 50% (Fig. 5*D*). We tested whether mice could categorize laser frequencies in a behavioral categorization task. That is, the lower frequencies (10, 12, and 14 Hz) were rewarded if mice went to the left port, and higher frequencies (16, 18, and 20 Hz) were rewarded if they went to the right port (Fig. 5*E*, ports were counterbalanced). We found that transgenic mice chose the “high” port more as the laser frequency increased (Fig. 5*F*). In contrast, intermediate frequencies (14 or 16 Hz) were more difficult to discriminate, possibly because of the similarity between the perceptual properties evoked by these two frequencies (Stutz et al., 1974). We conclude that mice could categorize distinct experiences induced by different optogenetic frequency parameters.

Movie 5. Frequency discrimination task. Transgenic mice had to discriminate between two laser stimulation frequencies. 10.1523/ENEURO.0216-22.2022.video.5

### Optoception does not require that the optogenetic stimulation be rewarding

**Figure 6.**
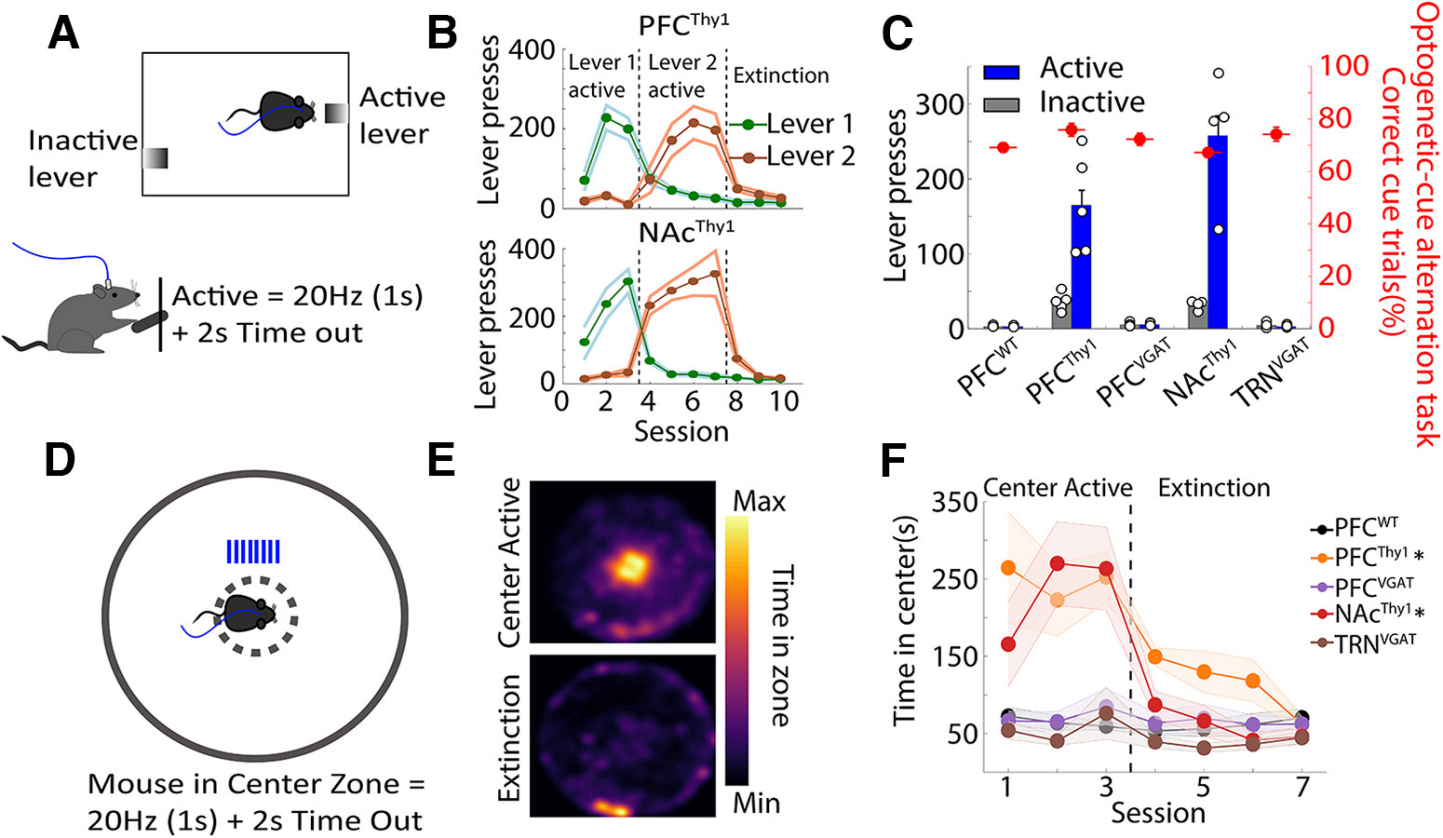
Optoception can guide behavior regardless of whether brain perturbations elicited rewarding effects or not. ***A***, Scheme of a lever self-stimulation task. Animals can trigger the delivery of laser stimulation by pressing the active lever (20 Hz, 1 s + 2 s of time out). The inactive lever was recorded but had no programmed consequence. ***B***, The number of lever presses across sessions. This shows that stimulation of PFC^Thy1^ and NAc^Thy1^ was rewarding, as indicated by the number of lever presses. After three sessions, the active lever was switched to inactive and tested for four additional sessions. Levers were counterbalanced across subjects. In the Extinction phase, both levers were Inactive, and thus no laser stimulation was evoked. Error bars indicate SEM. ***C***, Mean lever presses (excluding Extinction sessions). Small white dots indicate the number of mice tested. Overlapped also shows their average performance (right axis) achieved in the optogenetic-cue alternation task see solid red circles. ***D***, Open field center self-stimulation task. In this task, mice had to cross the center zone to receive laser stimulation (20 Hz, 1 s + 2 s of time out). Note that no other reward or stimuli were delivered. ***E***, A representative heat map of a PFC^Thy1^ mouse crosses the center (Active) to self-stimulate. The bottom panel shows an extinction session of the same mouse. ***F***, The time spent in the center zone across sessions for all groups; *p <* *0.05, two-way ANOVA, Dunnett *post hoc*, significantly differ from PFC^WT^ during active sessions. Extended Data Figure 6-1 shows that stimulation in PFC^VGAT^ or TRN^VGAT^ mice is not aversive.

It is well known that rats could guide behavior using the rewarding effects evoked by electrically stimulating the medial forebrain bundle (Wu et al., 2016). Hence, one possibility is that mice learn optoception because the optogenetic perturbation reinforced its behavior. To test this possibility, mice were trained in an operant self-stimulation task to demonstrate that the rewarding effects are important but not essential for optoception. In this task, mice press an active lever to trigger 1-s laser stimulation (Fig. 6*A*; Movie 6). Only PFC^Thy1^ and NAc^Thy1^ mice readily pressed the active lever, indicating that the stimulation was rewarding (Prado et al., 2016). In contrast, optogenetic stimulation was not rewarding for control PFC^WT^, PFC^VGAT^, and TRN^VGAT^ mice (Fig. 6*B*). Nevertheless, all groups performed equally well in the optogenetic-cue alternation task (Fig. 6*C*, red dots). Once more, the rewarding effects of these stimulations were confirmed in a real-time open field task, in which mice were optogenetically activated every time they crossed the center of the open field (Fig. 6*D*; Movie 7). As expected, WT mice rarely visited the center of the open field. In contrast, PFC^Thy1^ somas stimulation and activation of its glutamatergic afferent inputs into the NAc^Thy1^ (Prado et al., 2016) increased the time visiting the center when this zone triggered optogenetic self-stimulation but not during extinction sessions (Figs. 6*E*,*F*). In contrast, stimulation of GABAergic somas in both PFC^VGAT^ and TRN^VGAT^ mice seems to be neutral, i.e., no significant rewarding or aversive effects were observed (Extended Data Fig. 6-1). Thus, although not all stimulations assayed were rewarding, they all equally served as an optoceptive cue to guide behavior (Fig. 6*C*, red dots).

10.1523/ENEURO.0216-22.2022.f6-1 Extended Data Figure 6-1 Activation of GABAergic neurons in PFC or TRN was not aversive nor rewarding. ***A***, rtCPP. Mice were placed in the box with two different contexts (***A*** vs ***B***). Mice were stimulated on the less preferred side during three consecutive sessions, and finally, they were placed in a test session without stimulation. ***B***, Preference index in the side condition. Values above 0.5 mean that stimulation is preferred, while values below indicate that stimulation is avoided. PFC^VGAT^ and TRN^VGAT^ were not significantly different relative to PFC^WT^. Download Figure 6-1, TIF file.

Movie 6. Lever self-stimulation task. Example of NAc^Thy1^ mice in a self-stimulation task. 10.1523/ENEURO.0216-22.2022.video.6

Movie 7. Open field center self-stimulation. Example of PFC^Thy1^ mice crossing the center to self-stimulate. 10.1523/ENEURO.0216-22.2022.video.7

### Activating or silencing a single cell type both serve as optoceptive cue

**Figure 7.**
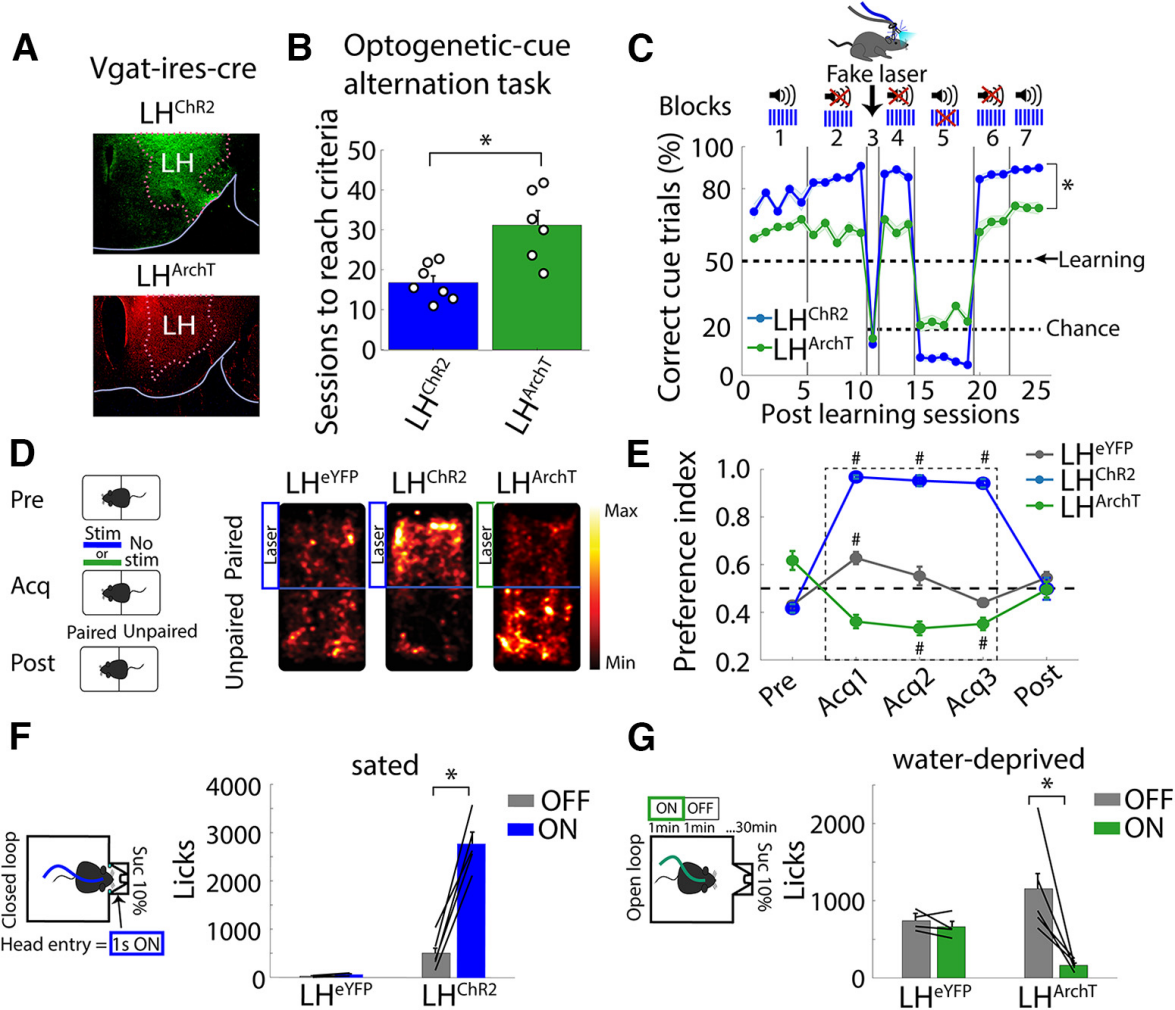
Mice could use both activation or silencing of a single cell type as a perceptible cue, although they evoked opposing behavioral effects on reward and feeding. ***A***, Histology of mice transfected with ChR2 or ArchT in Vgat-ires-cre mice (GABAergic neurons) of the LH (LH^ChR2^ or LH^ArchT^, respectively). ***B***, Sessions to reach learning criteria. Each dot represents a mouse; *p <* *0.001 unpaired *t* tests. ***C***, Correct trials in the presence of tone (2 kHz) and/or laser. Same conventions as in Figure 2*E*; *p <* *0.001 two-way ANOVA (transgenic mice × block). ***D***, Real-time Conditioned Place Preference (rtCPP). Left, rtCPP task consisted of three phases: pre-test (Pre, 1 session), acquisition (Acq, 3 sessions), and post-test (Post, 1 session). Right, Representative heat maps on the acquisition phase. Transfected Vgat-ires-cre mice with the enhanced yellow fluorescent protein (LH^eYFP^) were used as control. ***E***, Fraction of time spent on the paired side. Stimulation in LH^ChR2^ mice was rewarding (value > 0.5) while silencing in LH^ArchT^ was aversive (<0.5); #*p *<* *0.0001, ANOVA Dunnett *post hoc*, relative to pre-test. ***F***, Left, Schematic of the closed-loop task. Sated LH^ChR2^ or LH^eYFP^ mice were placed in a behavioral box with a sucrose sipper. Head entry into the port triggered optogenetic stimulation (1 s “on,” 20 Hz + 2 s time out, 473 nm). Right, total licks during the task. ***G***, Left panel, Open-loop task. In water-deprived LH^ArchT^ or control LH^eYFP^ mice, a continuous green laser was turned “on” in blocks of 1 min (532 nm) and 1 min with no-laser (“off”). Right, Total licks during the task; *p <* *0.001 paired *t* test. Extended Data Figure 7-1 depicts a raster plot of sucrose licking during stimulation of LH^ChR2^, LH^ArchT^, and LH^eYFP^ mice.

In another test to determine whether mice could perceive optogenetic stimuli, we explored whether they could learn optoception from activating or silencing a single cell type. This hypothesis was tested using the Vgat-ires-cre mice to drive the selective expression of ChR2 or Archaerhodopsin (ArchT) in GABAergic neurons in the lateral hypothalamus (LH) (Fig. 7*A*, LH^ChR2^ and LH^ArchT^, respectively). Thus, we could activate LH GABAergic neurons with ChR2 or silence them with the outward proton pump, ArchT (Chow et al., 2010). These mice could learn to use both the optogenetic activation and silencing of GABAergic neurons as a cue to solve the optogenetic-cue alternation task (Fig. 7*B*,*C*, block 1). However, stimulation of LH GABAergic neurons induced a faster (Fig. 7*B*, unpaired *t* test, *t*_(11)_ = 3.774, *p *<* *0.01) and better task performance than silencing them (Fig. 7*C*, two-way ANOVA, factor mice; *F*_(1,6)_ = 81.69, *p* < 0.0001; blocks *F*_(6,6)_ = 394.22, *p* < 0.0001 and the interaction mice × blocks, *F*_(6,305)_ = 35.4, *p *<* *0.0001). Moreover, these mice also maintained their task performance above chance level once the tone 2 kHz was removed from the combined cue (i.e., they received the laser only; Fig. 7*C*, blocks 2, 4, and 6). In contrast, their task performance dropped to chance level when they were tested with a “fake laser” (Fig. 7*C*, block 3) or when only the tone was delivered as a cue (Fig. 7*C*, block 5). Taken together, these results proved that these mice neglected the 2-kHz tone and used any sensation induced by optogenetic manipulations to guide behavior. LH GABAergic neurons were chosen because it is well established that their bidirectional activity caused opposing behavioral effects (Jennings et al., 2013; Nieh et al., 2016; Garcia et al., 2021). For example, in a rtCPP, we corroborated that bulk soma stimulation of GABAergic neurons (LH^ChR2^) is rewarding (Fig. 7*D*,*E*) in the sense that they preferred the side paired with the laser stimulation, whereas silencing them (LH^ArchT^) produced aversion since the mice avoided the side paired with the laser (Fig. 7*D*,*E*; Nieh et al., 2016). Opposing effects on feeding behavior were also elicited (Fig. 7*F*,*G*; Jennings et al., 2015; Siemian et al., 2021). In sated mice, stimulation of LH GABAergic neurons promoted consumption (Fig. 7*F*; LH^ChR2^), whereas in water-deprived mice, silencing them reduced sucrose intake (Fig. 7*G*, LH^ArchT^; Extended Data Fig. 7-1). Although activating (or silencing) LH GABAergic neurons had opposing effects on reward and feeding, these manipulations were also perceived and used as feedback cues to solve the optogenetic-cue alternation task.

10.1523/ENEURO.0216-22.2022.f7-1 Extended Data Figure 7-1 Activating or silencing of LH GABAergic neurons has opposing behavioral effects on feeding. ***A***, Schematic of closed-loop stimulation task. Sated LH^ChR2^ or LH^eYFP^ mice were placed in a behavioral box equipped with a sipper in a central port. The sipper was filled with sucrose 10%. In this task, the laser was triggered by a head entry in the central port (1 s, 20 Hz + 2 s time out, 473 nm). ***B***, Raster plot aligned to head entries for one LH^ChR2^ subject, red ticks = licks, blue ticks = laser. ***C***, Mean licks executed by LH^ChR2^ or LH^eYFP^ throughout sessions. The blue rectangle represents a laser session; the last three sessions were extinction sessions (no laser). ***D***, Schematics of the open-loop stimulation. Water-deprived LH^ArchT^ mice were located in a similar behavioral box to ***A***, but with blocks of 1 min “on,” 1 min “off” (continuous pulse, at 532 nm). ***E***, Upper panel, Rater plot of one LH^ArchT^ mouse, aligned to laser onset (time = 0), green rectangles indicate laser period, whereas red ticks indicate individual licks. Below is shown the PSTH average of lick responses across trials. ***F***, Upper panel, Histogram of each stimulation block in the control LH^eYFP^ mice. Below is a histogram of lick responses for LH^ArchT^ mice. Download Figure 7-1, TIF file.

### Optoception is not a generic sensation that can be generalized across regions, instead is a specific experience

**Figure 8.**
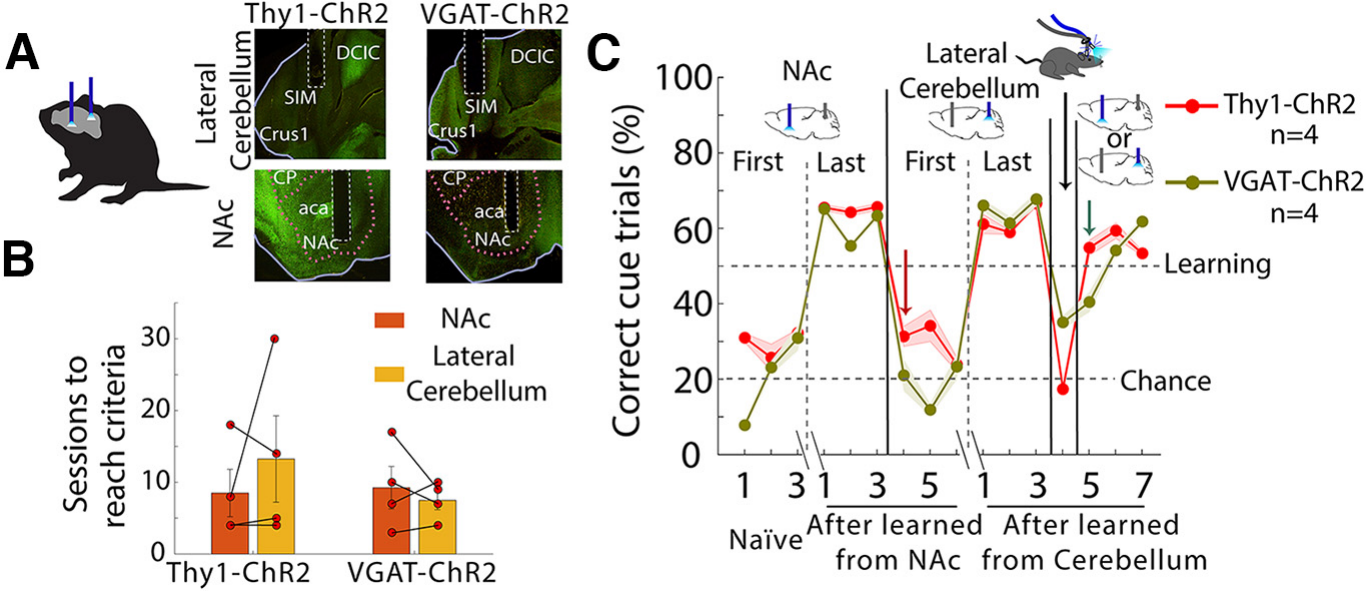
Optoception is not a generic sensation that can be generalized across regions, instead is a specific experience. ***A***, Left panel, Two optic fibers, one implanted in the NAc and the second in the lateral cerebellum from the same hemisphere. Right panel, Histology of implantation sites in the same two regions but now in the VGAT-ChR2 mice. ***B***, Sessions to reach learning criteria from the optogenetic cue-alternation task. Each dot represents a mouse. ***C***, Percent correct of cue trials; we plotted the first three and the last three sessions, in which mice first used optogenetic perturbations in the NAc to solve the task, and then it was switched to stimulation in the lateral cerebellum. In the first session, task performance dropped to chance with lateral cerebellum perturbation (see red arrow), suggesting that animals did not feel similar sensory qualia. Nevertheless, they can also learn to use lateral cerebellum perturbations to guide behavior after the training. In one session, some mice were tested in a Fake laser condition. Finally, we randomly interleaved stimulations from both brain regions within the same session. Surprisingly, after learning, they can indistinctly use randomly interleaved stimulations from both brain regions to guide behavior. Error bars indicate SEM.

The variability in the number of sessions needed for each mouse to reach the learning criteria (Figs. 2*D*, 3*A*, 5*C*, 7*B*) may reflect that each stimulation site induces a different experience, some easier to perceive than others. Then, it raises the possibility that optoception is a specific experience that cannot always generalize across regions. Thus, to test whether optogenetic perturbations of one brain region can generalize to a second not previously stimulated area, we trained Thy1-ChR2 and VGAT-ChR2 mice with fiber optics in both NAc and lateral cerebellum (Fig. 8*A*). We choose lateral cerebellum because they do not connect monosynaptically with the NAc, although they can disynaptically communicate (D’Ambra et al., 2021). Mice were trained in an optogenetic cue- alternation task (using the laser only). First, they received optogenetic perturbations in the NAc as the cue to solve the task (Fig. 8*B*). Once they reached the learning criteria (i.e., three sessions above 50% of correct cue trials), they received the optogenetic perturbation in the lateral cerebellum in the following session. We hypothesize that if optoception is based on a generic subjective experience, mice will generalize the sensation to other regions, and thus, task performance will not decrease. In contrast, to this prediction, we observed that the task performance drastically dropped to chance level (Fig. 8*C*, red arrow), suggesting that optoception does not rely on a generic experience. Nevertheless, with training, mice can also learn to use lateral cerebellum perturbations as an optoceptive cue. We found that task performance also decreased in a fake laser session, showing that mice did not merely use the light as a cue. Finally, we randomly interleaved stimulations from both brain regions within the same session. Impressively, the Thy1-ChR2 mice can indistinctly use interleaved stimulations from both brain regions to guide behavior, even from the first cerebellum stimulation day (see Fig. 8*C*, green arrow). Most likely, these mice use each specific sensation induced by each brain region to drive behavior. The VGAT-ChR2 mice needed an extra day to learn to use both perturbations to solve the task.

## Discussion

Our findings collectively reveal that mice were capable of perceiving arbitrary optogenetic perturbations. It was found that mice detected and actively reported activation and silencing of various cell types and brain regions. Mice could even sense a single laser pulse, discriminate, and categorize between distinct laser frequencies. Moreover, optoception occurred even when optogenetic activation or silencing of a single cell-type elicited rewarding or aversive interoceptive effects, or whether it promoted feeding or stopped it. We proposed that mice perceived the interoceptive state or sensation elicited by the optogenetically activated brain circuit and then learned to use it as a conditioned cue to guide behavior. This aligns well with the findings of Doty, 1965; who trained monkeys to report electrical stimulation by pressing a lever to obtain a reward and avoid an electric shock on the leg or tail (Doty, 1965), as well as those of Mazurek and Schieber (2017) who asked them to discriminate the parameter of intracranial stimulation delivered in premotor cortex (Mazurek and Schieber, 2017). In both cases, monkeys could detect when and whereinto the premotor cortex of the stimulation was delivered. Pioneering research using electrical (Houweling and Brecht, 2008; Fridman et al., 2010) or optogenetic (Huber et al., 2008) stimulation showed that rodents could also report a brief stimulation in the somatosensory barrel cortex by inducing an artificial sensory sensation. Likewise, electrical stimulation of the auditory thalamus or the visual pathway could serve as a cue to generate eyeblink conditioning (Campolattaro et al., 2007; Halverson et al., 2009; Halverson and Freeman, 2010). Our results confirm and further extend these observations to optogenetic manipulations most often employed, even in nonsensory cortical or subcortical areas and through activation or silencing of a single cell type. Given that the primary goal of optogenetics is to determine the physiological function (i.e., necessity and sufficiency) of a particular cell type support. This study’s results revealed an unexpected but important side effect evoked by the most performed optogenetic perturbations. Thus, special attention should be paid to the unintended perceptual properties induced by optogenetics.

This study proposed that if optogenetic activation is strong enough to perturb spiking homeostasis, it will induce an interoceptive signal or any sensory/motor discriminative stimulus that would make mice aware of the brain region that was either activated or silenced optogenetically (Berntson and Khalsa, 2021). Alternatively, recent research found that holographic optogenetic activation of <15 olfactory bulb neurons in synchronicity (but not asynchronous) is necessary for mice to detect optogenetic perturbations (Gill et al., 2020). Thus, E/I imbalance (Prado et al., 2016; Dalgleish et al., 2020) or synchronicity (Gill et al., 2020) may be important for perceiving neuronal perturbations.

How humans and mice experience brain manipulations, electrical or optogenetic, is an intriguing question (Mazurek and Schieber, 2019). Are they experienced as a natural or artificial stimulus? One can argue that co-activation of an arbitrarily large number of neurons rarely occurs under physiological conditions (with optogenetics, this effect is perhaps exacerbated since only one specific cell type and associated brain circuits are co-activated). Thus, it would likely be experienced as artificial (Huber et al., 2008; Mazurek and Schieber, 2019; Parvizi et al., 2022). However, more local intracranial microstimulation of the somatosensory cortex (S1) seems to be experienced more naturally since it could even be substituted for a natural sensory stimulus (Romo et al., 1998; Tabot et al., 2013). Of course, those studies could not rule out some degree of embodiment (Mazurek and Schieber, 2019). The seminal work of Wilder Penfield revealed that electrical stimulation could induce a “brainbow” of effects comprising noticeable movements, urge to move, somatosensory, visual, or auditory percepts, skin tingling or numbness, as well as rewarding or aversive effects (Olds, 1956), and even complex emotions (Penfield and Rasmussen, 1950). However, other instances produced no identifiable effect (Penfield and Rasmussen, 1950). Based on these results, in our experiments, it would be unlikely that mice felt precisely the same interoceptive (or any other sensory) sensation in each optogenetic manipulation assayed herein (Stutz et al., 1969). What is clear is that all transgenic mice acquired the task. However, some subjects (or stimulation sites) differed in task performance and required more sessions to achieve learning, suggesting that not all optogenetic manipulations were equally experienced. To this point, our generalization experiment between two regions confirmed that the sensation elicited by perturbing the NAc did not transfer to the lateral cerebellum (Fig. 8), suggesting that each stimulation most likely evoked a unique experience. In this regard, mice could also categorize low and high laser frequencies delivered into the same brain region (Fig. 5*F*), reflecting that different stimulation parameters are also experienced slightly differently. Therefore, different stimulation parameters may also evoke specific sensations. Of course, our data do not rule out the possibility that some brain regions, or less intense stimulation parameters, could not be perceived at all (Pan and Dudman, 2021). Nevertheless, our results demonstrate that some optogenetic manipulations are perceived. Thus, they may also be a helpful animal model to investigate specific interoceptive states evoked by various cell types (Berntson and Khalsa, 2021; Siemian et al., 2021), akin to the interoceptive conditioning phenomenon (Razran, 1961) elicited by drug-induced body states (Solinas et al., 2006; Bevins and Besheer, 2014; Ceunen et al., 2016). Our results also suggest that the rewarding, neutral, or aversive effects induced by optogenetic manipulations are not necessary to experience optoception, since mice learn to use all three interoceptive states as a discriminative stimulus (Stutz et al., 1974). We posit that mice would be aware of most, if not all, optogenetic brain perturbations, probably using interoception or any other sensory/motor stimuli evoked by the stimulation.

Our results support the idea that the brain is capable of “monitoring” its self-activity, perhaps via its evoked interoceptive state or discriminative stimuli, as previously suggested but not demonstrated by classic experiments of volitional control of neural signals proposed by Eberhard E. Fetz (Fetz, 1969), since these experiments necessarily require an exteroceptive sensory stimulus (i.e., auditory or visual) as a feedback cue to learn (Koralek et al., 2012).

The use of optoception also implies that it can be implemented as an independent sensory channel to control brain-computer interfaces. Prsa et al. (2017) recently showed that mice could use artificial optogenetic stimulation of the S1 cortex as sensory feedback to accelerate the control of their M1 neuronal activity (Prsa et al., 2017), thereby presenting an opportunity for using optoception as a parallel information channel to perform brain-computer interfaces (Koralek et al., 2012; Yadav et al., 2021). Our results extend these observations to show that the cortex or subcortical regions and stimulating or silencing a single cell type could be used as an additional sensory channel to introduce information to the brain.

Although our data suggest that mice experienced a “conscious” perception of optogenetic perturbations, an intriguing alternative explanation is that optogenetic perturbations might induce a Hebbian learning process of a nonconscious type (Lewicki et al., 1992; Lebedev and Ossadtchi, 2018), in which repeated paring of optostimulation with the activation (or silencing) of a particular neuronal circuit caused plasticity of synaptic weights and promoted the unconscious acquisition of information (Lebedev and Ossadtchi, 2018). Lebedev and Ossadtchi suggested this possibility as a mechanism for electrical stimulation. Thus, it is possible that Hebbian plasticity also contributed to the emergence and shaping of the animal’s conscious experiences caused by optogenetic stimulation. We quote, “Hebbian associative learning could eventually result in the emergence of realistic perceptions associated with such stimulation” (Lebedev and Ossadtchi, 2018). Further studies should explore whether optogenetic perturbations also elicit “unconscious” acquisition of information. Our findings that mice could also report optogenetic manipulations in the cerebellum, a brain region thought to play a minimal role in consciousness (Tononi and Koch, 2008; Clausi et al., 2017), support this idea.

Previous studies in the visual cortex suggest that mice readily detect only spike increments while equivalent decrements on V1 spiking impair performance in a visual contrast change detection task (Cone et al., 2020). In contrast, our findings uncovered that mice could readily perceive GABAergic neurons’ activation in multiple brain regions, including the silencing (using ArchT) of LH GABAergic neurons. Our findings are in agreement with previous studies reporting that “mice could reliably report optogenetic stimulation of VIP (vasoactive intestinal peptide) neurons in primary visual cortex V1 in the absence of a visual stimulus” (Cone et al., 2019). However, the same authors found that stimulation of other interneurons, the somatostatin (SST), and parvalbumin (PV) in V1 induced a perceptual impairment of visual information that affected the detection of visual contrast changes and task performance. Thus, they concluded that “mice could not learn to detect decrements in V1 spike rate even with extended practice.” This apparent discrepancy between Cone’s work and ours might be because they did not directly explore whether mice could use optogenetic stimulation of SST or PV only as a conditioned cue (Cone et al., 2020). Alternatively, this could reflect a peculiarity of V1 region where mice could not detect the activation of these interneurons because they deteriorate the processing of visual information. Further studies should more carefully dissect the contribution of optoception from the perceptual or cognitive function ascribed to the perturbed brain circuit (Histed et al., 2013).

The ability to manipulate the activity of genetically defined cell types via optogenetics has been a game-changing technology in neuroscience (Deisseroth, 2011). Given that the primary goal of optogenetics is to unveil the function of a specific cell type, our results highlight the importance of considering their unintended perceptual effects in interpreting optogenetic experiments, given that mice could also learn from brain stimulation per se.

## Synthesis

Reviewing Editor: Christine Portfors, Washington State University Decisions are customarily a result of the Reviewing Editor and the peer reviewers coming together and discussing their recommendations until a consensus is reached. When revisions are invited, a fact-based synthesis statement explaining their decision and outlining what is needed to prepare a revision will be listed below. The following reviewer(s) agreed to reveal their identity: NONE. Note: If this manuscript was transferred from JNeurosci and a decision was made to accept the manuscript without peer review, a brief statement to this effect will instead be what is listed below.

Thank you for thoroughly addressing the previous reviewers’ comments.

## References

[B1] Arenkiel BR, Peca J, Davison IG, Feliciano C, Deisseroth K, Augustine GJ, Ehlers MD, Feng G (2007) In vivo light-induced activation of neural circuitry in transgenic mice expressing channelrhodopsin-2. Neuron 54:205–218. 10.1016/j.neuron.2007.03.005 17442243 PMC3634585

[B2] Babl SS, Rummell BP, Sigurdsson T (2019) The spatial extent of optogenetic silencing in transgenic mice expressing channelrhodopsin in inhibitory interneurons. Cell Rep 29:1381–1395.e4. 10.1016/j.celrep.2019.09.049 31665647

[B3] Berntson GG, Khalsa SS (2021) Neural circuits of interoception. Trends Neurosci 44:17–28. 10.1016/j.tins.2020.09.011 33378653 PMC8054704

[B4] Bevins RA, Besheer J (2014) Interoception and learning: import to understanding and treating diseases and psychopathologies. ACS Chem Neurosci 5:624–631. 10.1021/cn5001028 25010473 PMC4140586

[B5] Campolattaro MM, Halverson HE, Freeman JH (2007) Medial auditory thalamic stimulation as a conditioned stimulus for eyeblink conditioning in rats. Learn Mem 14:152–159. 10.1101/lm.465507 17351138 PMC1838556

[B6] Carrillo-Reid L, Han S, Yang W, Akrouh A, Yuste R (2019) Controlling visually guided behavior by holographic recalling of cortical ensembles. Cell 178:447–457.e5. 10.1016/j.cell.2019.05.045 31257030 PMC6747687

[B7] Ceunen E, Vlaeyen JWS, Diest IV (2016) On the origin of interoception. Front Psychol 7:743. 10.3389/fpsyg.2016.00743 27242642 PMC4876111

[B8] Chow BY, Han X, Dobry AS, Qian X, Chuong AS, Li M, Henninger MA, Belfort GM, Lin Y, Monahan PE, Boyden ES (2010) High-performance genetically targetable optical neural silencing by light-driven proton pumps. Nature 463:98–102. 10.1038/nature08652 20054397 PMC2939492

[B9] Clausi S, Iacobacci C, Lupo M, Olivito G, Molinari M, Leggio M (2017) The role of the cerebellum in unconscious and conscious processing of emotions: a review. Appl Sci 7:521. 10.3390/app7050521

[B10] Cone JJ, Scantlen MD, Histed MH, Maunsell JHR (2019) Different inhibitory interneuron cell classes make distinct contributions to visual contrast perception. eNeuro 6:ENEURO.0337-18.2019. 10.1523/ENEURO.0337-18.2019 PMC6414440 30868104

[B11] Cone JJ, Bade ML, Masse NY, Page EA, Freedman DJ, Maunsell JHR (2020) Mice preferentially use increases in cerebral cortex spiking to detect changes in visual stimuli. J Neurosci 40:7902–7920. 10.1523/JNEUROSCI.1124-20.2020 32917791 PMC7548699

[B12] Craig AD (2002) How do you feel? Interoception: the sense of the physiological condition of the body. Nat Rev Neurosci 3:655–666. 10.1038/nrn894 12154366

[B13] Dalgleish HW, Russell LE, Packer AM, Roth A, Gauld OM, Greenstreet F, Thompson EJ, Häusser M (2020) How many neurons are sufficient for perception of cortical activity? Elife 9:e58889. 10.7554/eLife.58889 33103656 PMC7695456

[B14] D’Ambra AF, Jung SJ, Ganesan S, Antzoulatos EG, Fioravante D (2021) Cerebellar activation bidirectionally regulates nucleus accumbens medial shell and core. bioRxiv. doi: 10.1101/2020.09.28.283952.

[B15] Danskin B, Denman D, Valley M, Ollerenshaw D, Williams D, Groblewski P, Reid C, Olsen S, Blanche T, Waters J (2015) Optogenetics in mice performing a visual discrimination task: measurement and suppression of retinal activation and the resulting behavioral artifact. PLoS One 10:e0144760. 10.1371/journal.pone.0144760 26657323 PMC4686123

[B16] Dehghani N, Peyrache A, Telenczuk B, Le Van Quyen M, Halgren E, Cash SS, Hatsopoulos NG, Destexhe A (2016) Dynamic balance of excitation and inhibition in human and monkey neocortex. Sci Rep 6:23176. 10.1038/srep23176 26980663 PMC4793223

[B17] Deisseroth K (2011) Optogenetics. Nat Methods 8:26–29. 10.1038/nmeth.f.324 21191368 PMC6814250

[B18] Di Scala G, Mana MJ, Jacobs WJ, Phillips AG (1987) Evidence of Pavlovian conditioned fear following electrical stimulation of the periaqueductal grey in the rat. Physiol Behav 40:55–63. 10.1016/0031-9384(87)90185-5 3615655

[B19] Doty RW (1965) Conditioned reflexes elicited by electrical stimulation of the brain in macaques. J Neurophysiol 28:623–640. 10.1152/jn.1965.28.4.623 14347624

[B20] Fetz EE (1969) Operant conditioning of cortical unit activity. Science 163:955–958. 10.1126/science.163.3870.955 4974291

[B21] Fonseca E, de Lafuente V, Simon SA, Gutierrez R (2018) Sucrose intensity coding and decision-making in rat gustatory cortices. Elife 7:e41152. 10.7554/eLife.41152 30451686 PMC6292697

[B22] Fridman GY, Blair HT, Blaisdell AP, Judy JW (2010) Perceived intensity of somatosensory cortical electrical stimulation. Exp Brain Res 203:499–515. 10.1007/s00221-010-2254-y 20440610 PMC2875471

[B23] Garcia A, Coss A, Luis-Islas J, Puron-Sierra L, Luna M, Villavicencio M, Gutierrez R (2021) Lateral hypothalamic GABAergic neurons encode and potentiate sucrose’s palatability. Front Neurosci 14:608047. 33551725 10.3389/fnins.2020.608047 PMC7859279

[B24] Gill JV, Lerman GM, Zhao H, Stetler BJ, Rinberg D, Shoham S (2020) Precise holographic manipulation of olfactory circuits reveals coding features determining perceptual detection. Neuron 108:382–393.e5. 10.1016/j.neuron.2020.07.034 32841590 PMC8289117

[B25] Gil-Lievana E, Balderas I, Moreno-Castilla P, Luis-Islas J, McDevitt RA, Tecuapetla F, Gutierrez R, Bonci A, Bermúdez-Rattoni F (2020) Glutamatergic basolateral amygdala to anterior insular cortex circuitry maintains rewarding contextual memory. Commun Biol 3:139. 10.1038/s42003-020-0862-z 32198461 PMC7083952

[B26] Guo W, Hight AE, Chen JX, Klapoetke NC, Hancock KE, Shinn-Cunningham BG, Boyden ES, Lee DJ, Polley DB (2015) Hearing the light: neural and perceptual encoding of optogenetic stimulation in the central auditory pathway. Sci Rep 5:10319. 10.1038/srep10319 26000557 PMC4441320

[B27] Halverson HE, Freeman JH (2010) Medial auditory thalamic input to the lateral pontine nuclei is necessary for auditory eyeblink conditioning. Neurobiol Learn Mem 93:92–98. 10.1016/j.nlm.2009.08.008 19706335 PMC2815143

[B28] Halverson HE, Hubbard EM, Freeman JH (2009) Stimulation of the lateral geniculate, superior colliculus, or visual cortex is sufficient for eyeblink conditioning in rats. Learn Mem 16:300–307. 10.1101/lm.1340909 19395671 PMC2683004

[B29] Heffner HE, Heffner RS (2007) Hearing ranges of laboratory animals. J Am Assoc Lab Anim Sci 46:20–22. 17203911

[B30] Histed MH, Ni AM, Maunsell JHR (2013) Insights into cortical mechanisms of behavior from microstimulation experiments. Prog Neurobiol 103:115–130. 10.1016/j.pneurobio.2012.01.006 22307059 PMC3535686

[B31] Hölzl R, Erasmus L-P, Möltner A (1996) Detection, discrimination and sensation of visceral stimuli. Biol Psychol 42:199–214. 10.1016/0301-0511(95)05155-4 8770379

[B32] Houweling AR, Brecht M (2008) Behavioural report of single neuron stimulation in somatosensory cortex. Nature 451:65–68. 10.1038/nature06447 18094684

[B33] Huber D, Petreanu L, Ghitani N, Ranade S, Hromádka T, Mainen Z, Svoboda K (2008) Sparse optical microstimulation in barrel cortex drives learned behaviour in freely moving mice. Nature 451:61–64. 10.1038/nature06445 18094685 PMC3425380

[B34] Jennings JH, Rizzi G, Stamatakis AM, Ung RL, Stuber GD (2013) The inhibitory circuit architecture of the lateral hypothalamus orchestrates feeding. Science 341:1517–1521. 10.1126/science.1241812 24072922 PMC4131546

[B35] Jennings JH, Ung RL, Resendez SL, Stamatakis AM, Taylor JG, Huang J, Veleta K, Kantak PA, Aita M, Shilling-Scrivo K, Ramakrishnan C, Deisseroth K, Otte S, Stuber GD (2015) Visualizing hypothalamic network dynamics for appetitive and consummatory behaviors. Cell 160:516–527. 10.1016/j.cell.2014.12.026 25635459 PMC4312416

[B36] Khalsa SS, Lapidus RC (2016) Can interoception improve the pragmatic search for biomarkers in psychiatry? Front Psychiatry 7:121. 27504098 10.3389/fpsyt.2016.00121 PMC4958623

[B37] Koralek AC, Jin X, Long JD 2nd, Costa RM, Carmena JM (2012) Corticostriatal plasticity is necessary for learning intentional neuroprosthetic skills. Nature 483:331–335. 10.1038/nature10845 22388818 PMC3477868

[B38] Kumar S, Black SJ, Hultman R, Szabo ST, DeMaio KD, Du J, Katz BM, Feng G, Covington HE, Dzirasa K (2013) Cortical control of affective networks. J Neurosci 33:1116–1129. 10.1523/JNEUROSCI.0092-12.2013 23325249 PMC3711588

[B39] Lebedev MA, Ossadtchi A (2018) Commentary: injecting instructions into premotor cortex. Front Cell Neurosci 12:65. 10.3389/fncel.2018.00065 29637931 PMC5880917

[B40] Lewicki P, Hill T, Czyzewska M (1992) Nonconscious acquisition of information. Am Psychol 47:796–801. 10.1037/0003-066x.47.6.796 1616179

[B41] Maffei A, Fontanini A (2009) Network homeostasis: a matter of coordination. Curr Opin Neurobiol 19:168–173. 10.1016/j.conb.2009.05.012 19540746 PMC3427905

[B42] Marshel JH, Kim YS, Machado TA, Quirin S, Benson B, Kadmon J, Raja C, Chibukhchyan A, Ramakrishnan C, Inoue M, Shane JC, McKnight DJ, Yoshizawa S, Kato HE, Ganguli S, Deisseroth K (2019) Cortical layer–specific critical dynamics triggering perception. Science 365:eaaw5202. 10.1126/science.aaw5202 31320556 PMC6711485

[B43] Mazurek KA, Schieber MH (2017) Injecting instructions into premotor cortex. Neuron 96:1282–1289.e4. 10.1016/j.neuron.2017.11.006 29224724 PMC5739962

[B44] Mazurek KA, Schieber MH (2019) How is electrical stimulation of the brain experienced, and how can we tell? Selected considerations on sensorimotor function and speech. Cogn Neuropsychol 36:103–116. 10.1080/02643294.2019.1609918 31076014 PMC6744321

[B45] Nieh EH, Vander Weele CM, Matthews GA, Presbrey KN, Wichmann R, Leppla CA, Izadmehr EM, Tye KM (2016) Inhibitory input from the lateral hypothalamus to the ventral tegmental area disinhibits dopamine neurons and promotes behavioral activation. Neuron 90:1286–1298. 10.1016/j.neuron.2016.04.035 27238864 PMC4961212

[B46] O’Connor DH, Hires SA, Guo ZV, Li N, Yu J, Sun QQ, Huber D, Svoboda K (2013) Neural coding during active somatosensation revealed using illusory touch. Nat Neurosci 16:958–965. 10.1038/nn.3419 23727820 PMC3695000

[B47] Olds J (1956) Pleasure centers in the brain. Sci Am 195:105–117. 10.1038/scientificamerican1056-105

[B48] Owen SF, Liu MH, Kreitzer AC (2019) Thermal constraints on in vivo optogenetic manipulations. Nat Neurosci 22:1061–1065. 10.1038/s41593-019-0422-3 31209378 PMC6592769

[B49] Pan WX, Dudman JT (2021) Phasic responses of mesolimbic dopamine neurons are recruited in parallel to sensorimotor pathways mediating cued approach (SSRN Scholarly Paper No. ID 3845000). Rochester, NY: Social Science Research Network.

[B50] Parvizi J, Veit MJ, Barbosa DAN, Kucyi A, Perry C, Parker JJ, Shivacharan RS, Chen F, Yih J, Gross JJ, Fisher R, McNab JA, Falco-Walter J, Halpern CH (2022) Complex negative emotions induced by electrical stimulation of the human hypothalamus. Brain Stimul 15:615–623. 10.1016/j.brs.2022.04.008 35413481

[B51] Pendharkar AV, Smerin D, Gonzalez L, Wang EH, Levy S, Wang S, Ishizaka S, Ito M, Uchino H, Chiang T, Cheng MY, Steinberg GK (2021) Optogenetic stimulation reduces neuronal nitric oxide synthase expression after stroke. Transl Stroke Res 12:347–356. 10.1007/s12975-020-00831-y 32661768 PMC7925487

[B52] Penfield W, Rasmussen T (1950) The cerebral cortex of man: a clinical study of localization of function. New York: Macmillian.

[B53] Porrero C, Rubio-Garrido P, Avendaño C, Clascá F (2010) Mapping of fluorescent protein-expressing neurons and axon pathways in adult and developing Thy1-eYFP-H transgenic mice. Brain Res 1345:59–72. 10.1016/j.brainres.2010.05.061 20510892

[B54] Prado L, Luis-Islas J, Sandoval OI, Puron L, Gil MM, Luna A, Arias-García MA, Galarraga E, Simon SA, Gutierrez R (2016) Activation of glutamatergic fibers in the anterior NAc shell modulates reward activity in the aNAcSh, the lateral hypothalamus, and medial prefrontal cortex and transiently stops feeding. J Neurosci 36:12511–12529. 10.1523/JNEUROSCI.1605-16.2016 27974611 PMC6705665

[B55] Prsa M, Galiñanes GL, Huber D (2017) Rapid integration of artificial sensory feedback during operant conditioning of motor cortex neurons. Neuron 93:929–939.e6. 10.1016/j.neuron.2017.01.023 28231470 PMC5330804

[B56] Razran G (1961) The observable and the inferable conscious in current Soviet psychophysiology: interoceptive conditioning, semantic conditioning, and the orienting reflex. Psychol Rev 68:1–147. 10.1037/h0039848 13740033

[B57] Romo R, Hernández A, Zainos A, Salinas E (1998) Somatosensory discrimination based on cortical microstimulation. Nature 392:387–390. 10.1038/32891 9537321

[B58] Sachidhanandam S, Sreenivasan V, Kyriakatos A, Kremer Y, Petersen CCH (2013) Membrane potential correlates of sensory perception in mouse barrel cortex. Nat Neurosci 16:1671–1677. 10.1038/nn.3532 24097038

[B59] Siemian JN, Arenivar MA, Sarsfield S, Aponte Y (2021) Hypothalamic control of interoceptive hunger. Curr Biol 31:3797–3809.e5. 10.1016/j.cub.2021.06.048 34273280 PMC8440483

[B60] Sippy T, Lapray D, Crochet S, Petersen CCH (2015) Cell-type-specific sensorimotor processing in striatal projection neurons during goal-directed behavior. Neuron 88:298–305. 10.1016/j.neuron.2015.08.039 26439527 PMC4622932

[B61] Sofroniew NJ, Vlasov YA, Hires SA, Freeman J, Svoboda K (2015) Neural coding in barrel cortex during whisker-guided locomotion. Elife 4:e12559. 10.7554/eLife.12559 26701910 PMC4764557

[B62] Solinas M, Panlilio LV, Justinova Z, Yasar S, Goldberg SR (2006) Using drug-discrimination techniques to study the abuse-related effects of psychoactive drugs in rats. Nat Protoc 1:1194–1206. 10.1038/nprot.2006.167 17406402

[B63] Stutz RM, Butcher RE, Rossi R (1969) Stimulus properties of reinforcing brain shock. Science 163:1081–1082. 10.1126/science.163.3871.1081 5764875

[B64] Stutz RM, Rossi RR, Hastings L, Brunner RL (1974) Discriminability of intracranial stimuli: the role of anatomical connectedness. Physiol Behav 12:69–73. 10.1016/0031-9384(74)90069-9 4589470

[B65] Tabot GA, Dammann JF, Berg JA, Tenore FV, Boback JL, Vogelstein RJ, Bensmaia SJ (2013) Restoring the sense of touch with a prosthetic hand through a brain interface. Proc Natl Acad Sci U S A 110:18279–18284. 10.1073/pnas.1221113110 24127595 PMC3831459

[B66] Thomson EE, Carra R, Nicolelis MAL (2013) Perceiving invisible light through a somatosensory cortical prosthesis. Nat Commun 4:1482. 10.1038/ncomms2497 23403583 PMC3674834

[B67] Tononi G, Koch C (2008) The neural correlates of consciousness. Ann N Y Acad Sci 1124:239–261. 10.1196/annals.1440.004 18400934

[B68] Verrier R, Calvert A, Lown B (1975) Effect of posterior hypothalamic stimulation on ventricular fibrillation threshold. Am J Physiol 228:923–927. 10.1152/ajplegacy.1975.228.3.923 1115257

[B69] Vetere G, Tran LM, Moberg S, Steadman PE, Restivo L, Morrison FG, Ressler KJ, Josselyn SA, Frankland PW (2019) Memory formation in the absence of experience. Nat Neurosci 22:933–940. 10.1038/s41593-019-0389-0 31036944 PMC7592289

[B70] Vong L, Ye C, Yang Z, Choi B, Chua S, Lowell BB (2011) Leptin action on GABAergic neurons prevents obesity and reduces inhibitory tone to POMC neurons. Neuron 71:142–154. 10.1016/j.neuron.2011.05.028 21745644 PMC3134797

[B71] Wimmer RD, Schmitt LI, Davidson TJ, Nakajima M, Deisseroth K, Halassa MM (2015) Thalamic control of sensory selection in divided attention. Nature 526:705–709. 10.1038/nature15398 26503050 PMC4626291

[B72] Wu Z, Zheng N, Zhang S, Zheng X, Gao L, Su L (2016) Maze learning by a hybrid brain-computer system. Sci Rep 6:31746. 10.1038/srep31746 27619326 PMC5020320

[B73] Yadav AP, Li S, Krucoff MO, Lebedev MA, Abd-El-Barr MM, Nicolelis MAL (2021) Generating artificial sensations with spinal cord stimulation in primates and rodents. Brain Stimul 14:825–836. 10.1016/j.brs.2021.04.024 34015518 PMC8316418

[B74] Zhao S, Ting JT, Atallah HE, Qiu L, Tan J, Gloss B, Augustine GJ, Deisseroth K, Luo M, Graybiel AM, Feng G (2011) Cell type–specific channelrhodopsin-2 transgenic mice for optogenetic dissection of neural circuitry function. Nat Methods 8:745–752. 10.1038/nmeth.1668 21985008 PMC3191888

