# Supplementary figures and images for: Optoception: Perception of Optogenetic Brain Perturbations
Source: eNeuro. 2022 Jun 27;9(3):ENEURO.0216-22.2022. doi: 10.1523/ENEURO.0216-22.2022 (PMC9241931; doi:10.1523/ENEURO.0216-22.2022)

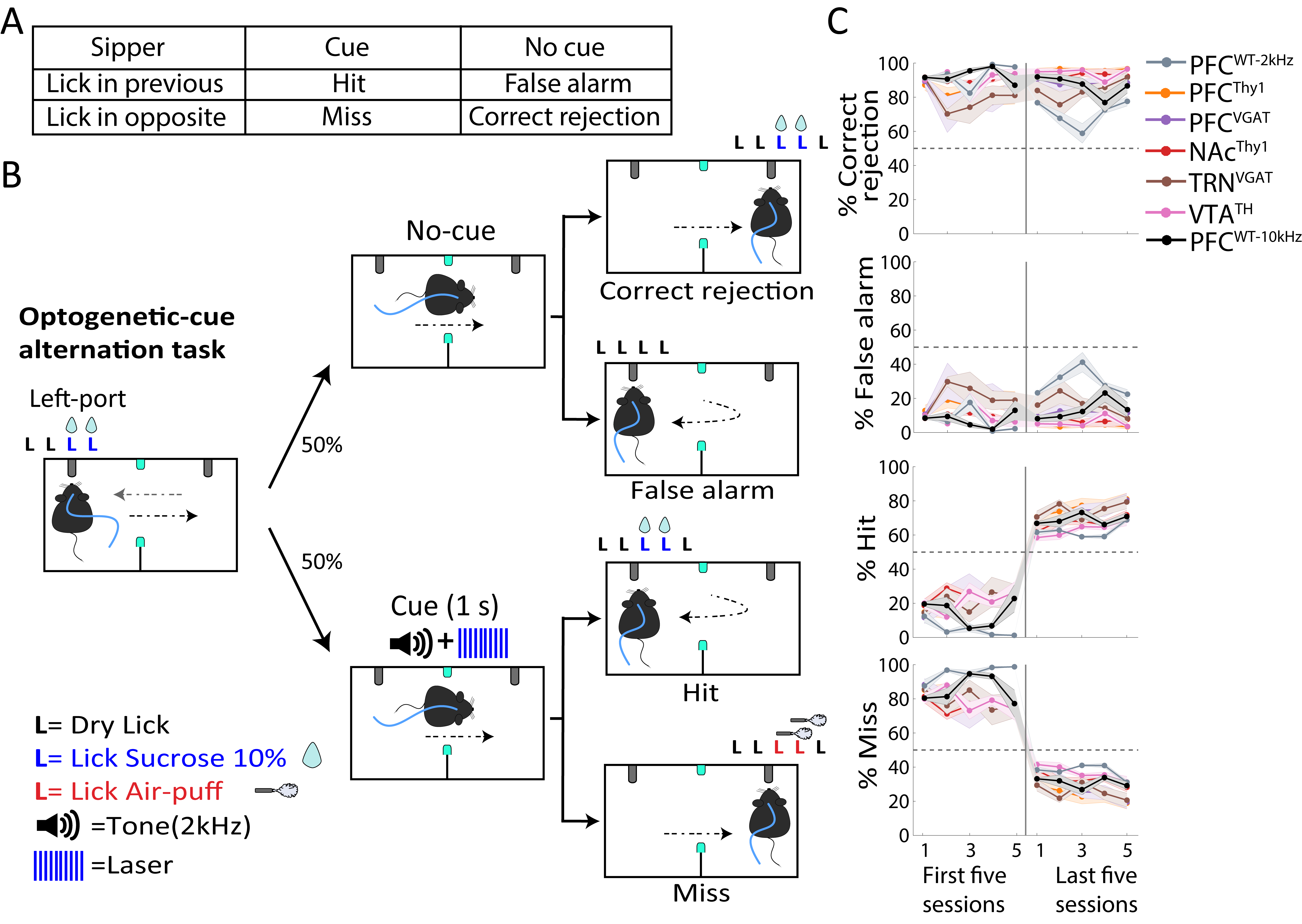

Supplement: Extended Data Figure 2-1 — Mice learn to return to the previously rewarded port only when the optogenetic cue is present (Hit trials). A, Table of different types of trials Correct Rejection, False Alarm, Hits, and Misses. In our task, mice had to continue alternating between sippers in no-cue trials (Correct Rejections), and very few False Alarm responses were observed. In contrast, in cue trials, lick responses were given in the previously rewarded sipper (Hits), and a few Misses were made. B, Schematic of the optogenetic-cue sipper alternation task separated by trial type. C, Task performance in the first five and last five sessions during the task. After reaching the learning criteria (horizontal dashed line), only hit trials increased while misses decreased. Correct rejection and False alarm maintained the same task performance as in the first five sessions. Error bars indicate SEM. Download Figure 2-1, TIF file. [file enu-eN-CFN-0216-22-s08.tif]

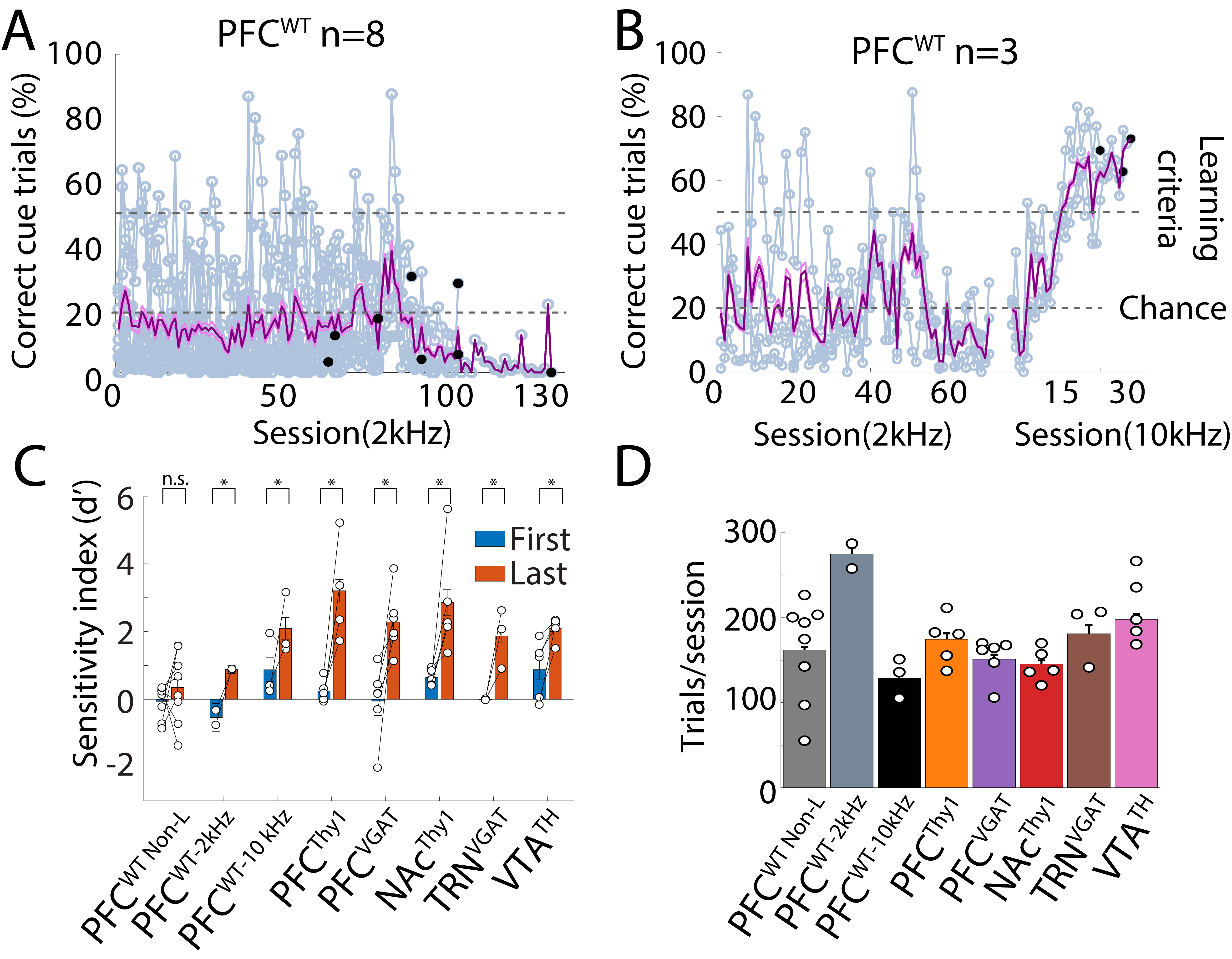

Supplement: Extended Data Figure 2-2 — The 2-kHz tone was less efficient than the optogenetic stimulation as a cue. A, Correct trials of PFCWT mice that did not reach the learning criteria trained at 2 kHz in the Optogenetic-cue sipper alternation task. Individual mice are shown in gray, and the mean ± SEM is shown in purple. Black dots showed the last session of each subject. B, The tone frequency was changed to 10 kHz. The task performance of the three nonlearners subjects, from panel A. The same subjects were also trained with a 10-kHz tone. Note that after the tone was changed from 2 to 10 kHz, they rapidly learned the task (gray dash line learning criteria). C, Sensitivity index (d prime, d’) was computed for the first and last five sessions. Transgenic mice showed values above d’ > 1. In contrast, PFCWT-2KHz exhibited values below d’ < 1, indicating that although they reached the learning criteria, they could not detect the cue as efficiently as transgenic mice or PFCWT-10kHz. PFCWT Non-L refers to nonlearners. D, Total trials (Cue + No-Cue). Each dot represents an individual subject; *p < 0.05 paired t test. Download Figure 2-2, TIF file. [file enu-eN-CFN-0216-22-s09.tif]

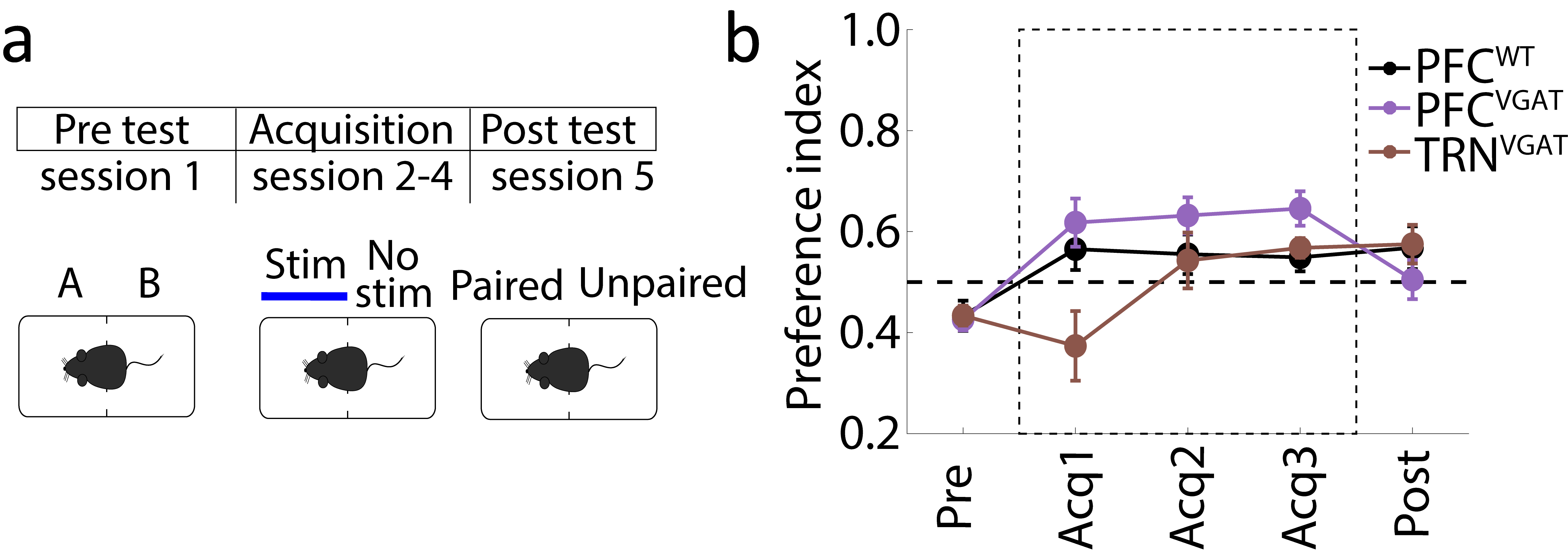

Supplement: Extended Data Figure 6-1 — Activation of GABAergic neurons in PFC or TRN was not aversive nor rewarding. A, rtCPP. Mice were placed in the box with two different contexts (A vs B). Mice were stimulated on the less preferred side during three consecutive sessions, and finally, they were placed in a test session without stimulation. B, Preference index in the side condition. Values above 0.5 mean that stimulation is preferred, while values below indicate that stimulation is avoided. PFCVGAT and TRNVGAT were not significantly different relative to PFCWT. Download Figure 6-1, TIF file. [file enu-eN-CFN-0216-22-s10.tif]

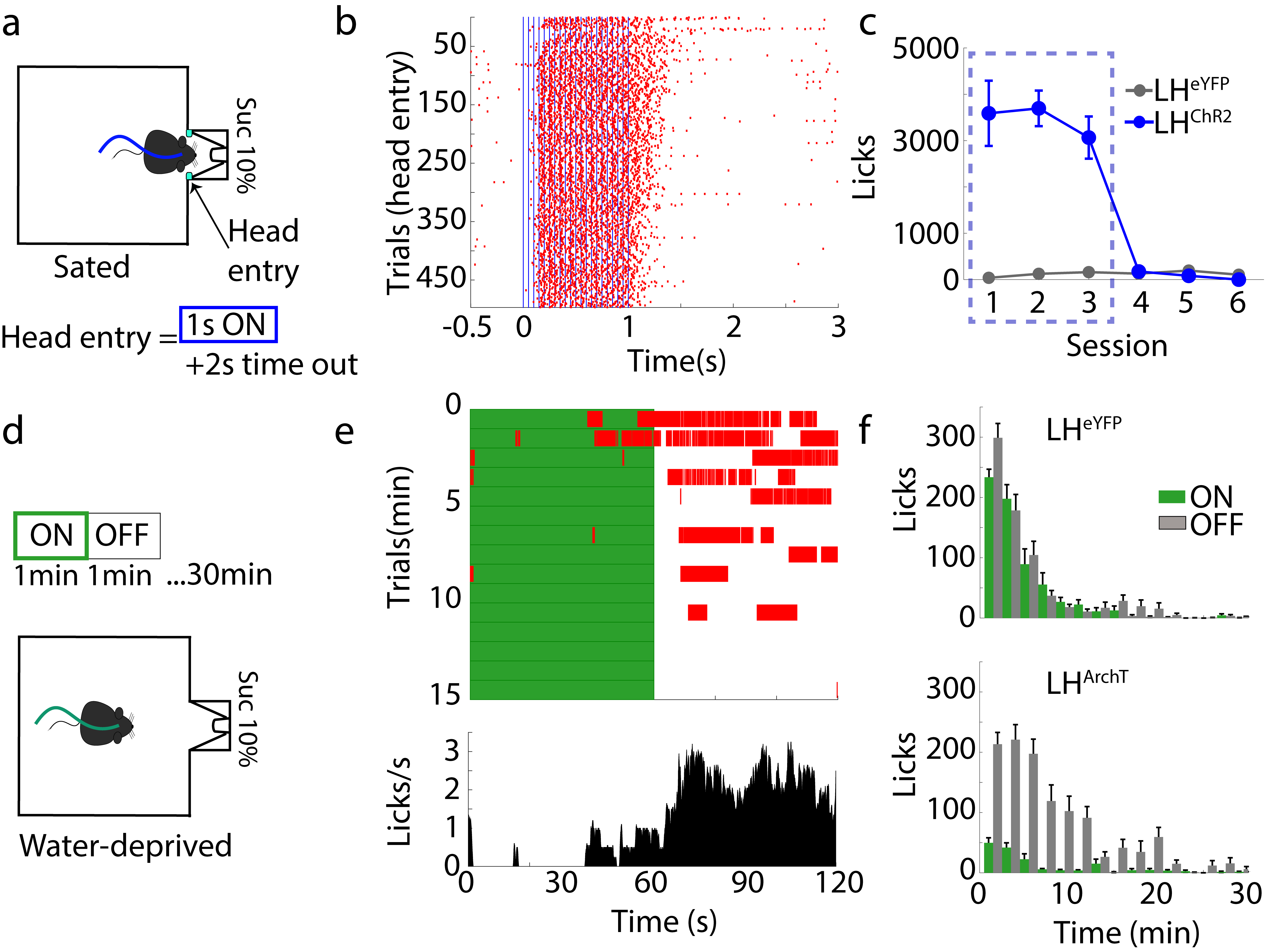

Supplement: Extended Data Figure 7-1 — Activating or silencing of LH GABAergic neurons has opposing behavioral effects on feeding. A, Schematic of closed-loop stimulation task. Sated LHChR2 or LHeYFP mice were placed in a behavioral box equipped with a sipper in a central port. The sipper was filled with sucrose 10%. In this task, the laser was triggered by a head entry in the central port (1 s, 20 Hz + 2 s time out, 473 nm). B, Raster plot aligned to head entries for one LHChR2 subject, red ticks = licks, blue ticks = laser. C, Mean licks executed by LHChR2 or LHeYFP throughout sessions. The blue rectangle represents a laser session; the last three sessions were extinction sessions (no laser). D, Schematics of the open-loop stimulation. Water-deprived LHArchT mice were located in a similar behavioral box to A, but with blocks of 1 min “on,” 1 min “off” (continuous pulse, at 532 nm). E, Upper panel, Rater plot of one LHArchT mouse, aligned to laser onset (time = 0), green rectangles indicate laser period, whereas red ticks indicate individual licks. Below is shown the PSTH average of lick responses across trials. F, Upper panel, Histogram of each stimulation block in the control LHeYFP mice. Below is a histogram of lick responses for LHArchT mice. Download Figure 7-1, TIF file. [file enu-eN-CFN-0216-22-s11.tif]
